# Co-option of immune effectors by the hormonal signalling system triggering metamorphosis in *Drosophila melanogaster*

**DOI:** 10.1371/journal.pgen.1009916

**Published:** 2021-11-29

**Authors:** Catarina Nunes, Takashi Koyama, Élio Sucena

**Affiliations:** 1 Evolution and Development Laboratory, Instituto Gulbenkian de Ciência, Oeiras, Portugal; 2 Section for Cell and Neurobiology, Department of Biology, University of Copenhagen, Copenhagen, Denmark; 3 Departamento de Biologia Animal, Faculdade de Ciências, Universidade de Lisboa, Lisbon, Portugal; Universidad de Valparaiso, CHILE

## Abstract

Insect metamorphosis is triggered by the production, secretion and degradation of 20-hydroxyecdysone (ecdysone). In addition to its role in developmental regulation, increasing evidence suggests that ecdysone is involved in innate immunity processes, such as phagocytosis and the induction of antimicrobial peptide (AMP) production. AMP regulation includes systemic responses as well as local responses at surface epithelia that contact with the external environment. At pupariation, *Drosophila melanogaster* increases dramatically the expression of three AMP genes, *drosomycin (drs)*, *drosomycin-like 2 (drsl2)* and *drosomycin-like 5 (drsl5)*. We show that the systemic action of *drs* at pupariation is dependent on ecdysone signalling in the fat body and operates via the ecdysone downstream target, *Broad*. In parallel, ecdysone also regulates local responses, specifically through the activation of *drsl2* expression in the gut. Finally, we confirm the relevance of this ecdysone dependent AMP expression for the control of bacterial load by showing that flies lacking *drs* expression in the fat body have higher bacterial persistence over metamorphosis. In contrast, local responses may be redundant with the systemic effect of *drs* since reduction of ecdysone signalling or of *drsl2* expression has no measurable negative effect on bacterial load control in the pupa. Together, our data emphasize the importance of the association between ecdysone signalling and immunity using *in vivo* studies and establish a new role for ecdysone at pupariation, which impacts developmental success by regulating the immune system in a stage-dependent manner. We speculate that this co-option of immune effectors by the hormonal system may constitute an anticipatory mechanism to control bacterial numbers in the pupa, at the core of metamorphosis evolution.

## Author summary

Developmental transitions in insects are regulated by the hormone ecdysone. Triggering metamorphosis constitutes a key feature of holometabolan insects and its evolution has required the establishment of new cross-talks between multiple organ systems and processes. Before metamorphosis, concomitantly with the initiation of a dramatic remodelling of larval tissues including the bacteria-containing gut, *D*. *melanogaster* increases the expression of three antimicrobial peptides (AMPs). In this study, we establish that such peak of antimicrobial peptide expression is part of the ecdysone pulse-dependent trigger of the metamorphic moult. We show that this immune response is deployed both locally and systemically, the latter being vital for the control of bacterial numbers in the pupal case. We establish a new role for ecdysone in the co-option of immunity by the metamorphosis programme and speculate that it may constitute an important element in the evolution of this developmental novelty.

## Introduction

Hormonal control of insect development is mainly dependent on the steroid hormone, 20-hydroxyecdsyone (hereafter, ecdysone) [1,2]. In vertebrates, steroid hormones and their nuclear receptors are key regulators of systemic immune responses, namely through the enhancement of inflammation [3]. Similarly, in insects, steroid hormones are also known to be involved in innate immunity [4–14]. Ecdysone titre is tightly regulated to control developmental transitions and as such, steroid hormone-dependent activation seems appropriate for stage-specific regulation of innate immunity [7,11–14].

When in contact with a microorganism, insects rely on the activation of three tightly related immunity processes: i) cellular immunity (i.e. phagocytosis and encapsulation of invading microorganisms); ii) the induction of proteolytic cascades, that culminate in melanisation; and iii) production of antimicrobial peptides (AMPs) [15,16]. In *Drosophila melanogaster*, AMP expression is primarily regulated at the transcriptional level through two distinct signalling pathways, Toll and Imd, triggered by sensing of Lys-type peptidoglycan and β-1,3-glucan or meso-diaminopimelic acid (DAP)-type peptidoglycan, respectively [15–18]. In insects with complete metamorphosis (holometabolans), AMP genes are usually silent in the absence of an immune challenge and their expression is induced upon injury and/or infection [18,19]. However, growing evidence suggests that AMPs may be up-regulated at specific developmental stages irrespective of pathogen presence.

Also, studies in the moth *Manduca sexta* show that expression of lysozyme and of the AMPs *cecropin A*, *cecropin B* and *hemolin* increases dramatically during metamorphosis, although it is not clear what regulates this increase and its role(s) *in vivo* [20–22]. This transcriptional AMP peak occurs before the end of histolysis of the larval midgut, that is, before cells are potentially exposed to any pathogen or bacterium. In this context, the accumulation of AMPs at the end of the feeding stage may constitute a first response against the possible harmful effects to the pupa of expanding bacteria carried-over from the gut remodelling process [23–26]. Nonetheless, studies in dipterans show that some species of bacteria in the larval gut can persist inside the host throughout metamorphosis [25–28]. In *Drosophila melanogaster*, RNAseq analysis revealed a peak in the expression of AMPs at pupariation, of which around 95% consists of three AMPs: *drosomycin (drs)*, *drosomycin-like 2 (drsl2)* and *drosomycin-like 5 (drsl5)* [29]. Furthermore, five different AMPs (*diptericin*, *drs*, *attacin-A*, *metchnikowin* and *cecropin A1*) are shown to contain putative *cis*-regulatory elements for potential binding of the functional ecdysone receptor complex, suggesting an ecdysone-dependent control of their expression [30]. Importantly, studies in the mosquito *Anopheles gambiae* have implicated ecdysone signalling in the regulation of immune genes following blood meals, a moment particularly prone to exposure to blood-borne pathogens [6,14]. Furthermore, the compelling association between ecdysone signalling and immunity at metamorphosis is not restricted to the humoral arm of the immune response. An ecdysone-dependent increase in phagocytic capacity of plasmatocytes at pupariation has been described and hypothesized to be particularly important given the higher predisposition for infection at the pupal stage [7].

Considering the above, we hypothesized that ecdysone signalling controlling developmental transitions was co-opted to regulate the expression of AMPs at pupariation. Previous *in vitro* study has shown that ecdysone promotes humoral immunity by increasing the expression of AMP genes, such as *diptericin*, *cecropin* and *attacin*, via the Ecdysone receptor-Ultraspiracle (EcR-USP) receptor complex in S2 cells [5]. In addition, a microarray analysis of 20-hydroxyecdysone-treated S2 cells revealed the existence of two distinct mechanisms for ecdysone-regulated AMP expression: one in which the critical step is the activation of peptidoglycan recognition protein (PGRP)-LC—a critical pattern recognition receptor (PRR) of the Imd pathway—by ecdysone for controlling the expression of *cecropin A1*, *attacin-A and defensin*; and another, in which the hormonally-controlled mechanism is absolutely required for the expression of *drs*, *metchnikowin* and *diptericin* [4]. However, the importance of the requirement of ecdysone for the expression of AMPs, and its role, nor the regulatory mechanism through which this hormone affects innate immunity have been explored.

In this study, we demonstrate that ecdysone regulates the expression of AMPs *in vivo* at pupariation, both systemically (by regulating the expression of *drs*) and locally in the midgut (by regulating the expression of *drsl2*). Importantly, the systemic expression of *drs* has an impact on the number of bacteria that persists and proliferates during metamorphosis, corroborating the hypothesis that the AMP peak has a function in controlling bacteria during metamorphosis. Furthermore, we show that this association between the expression of *drs* and ecdysone in the absence of infection occurs solely at pupariation and through the early ecdysone-response gene *Broad*, which is only expressed at the end of the feeding stage [31]. Taken together, our findings fill a gap in current knowledge by shedding light onto the mechanisms and biological significance of hormonal control of humoral immunity at metamorphosis with potential evolutionary importance in the emergence of holometaboly.

## Results

### *drs*, *drsl2* and *drsl5* are expressed at pupariation in a bacteria-independent manner

**Fig 1:**
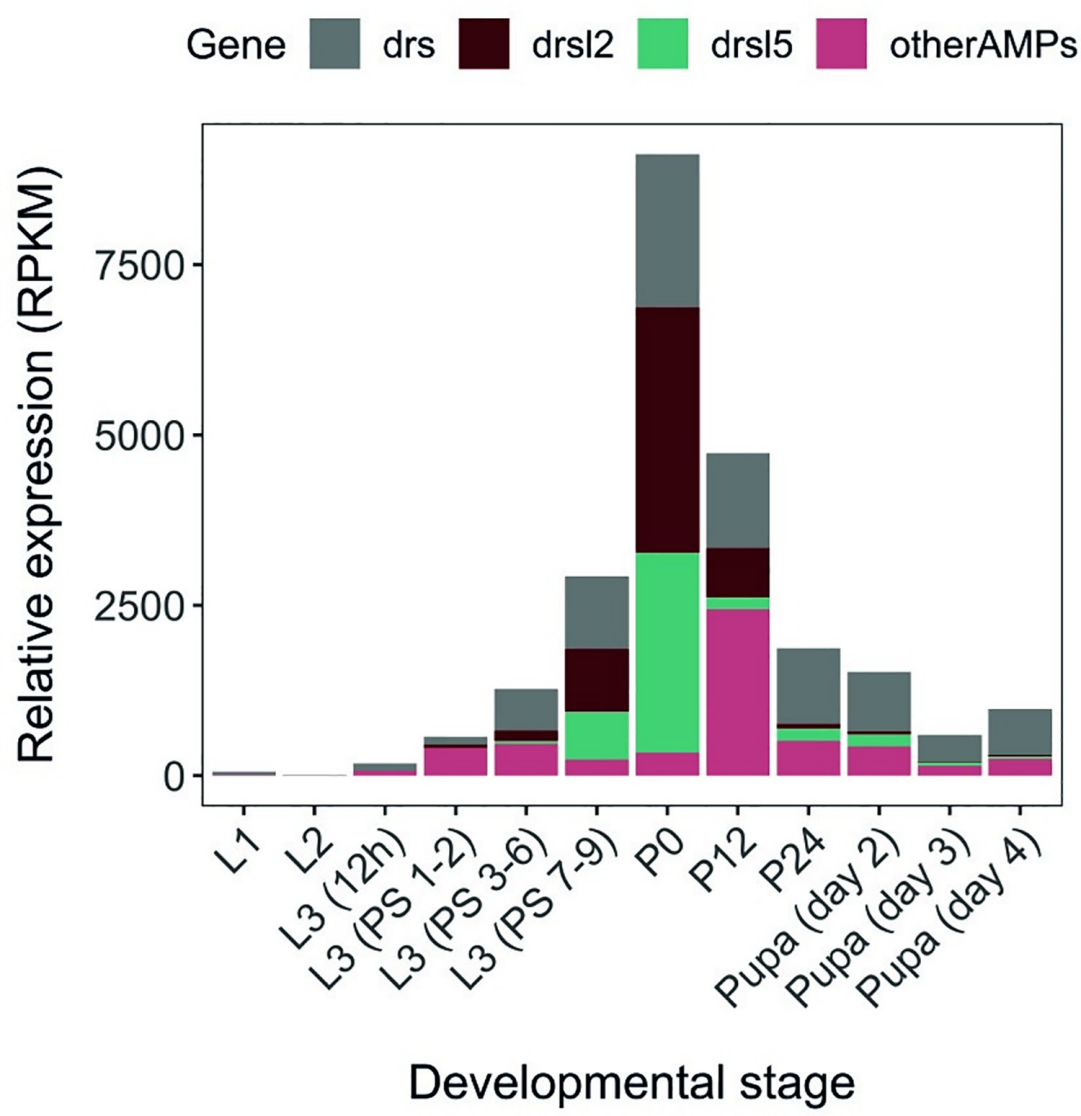
Developmental time course of AMP expression. The expression levels (RPKM) of *drs*, *drsl2*, *drsl5*, and all other AMPs as a single class are represented between L1 and 4-day-old pupae. An expression peak at P0 composed of *drs*, *drsl2*, *drsl5 is apparent*. Expression refers to whole body and is normalized in RPKM (Reads Per Kilobase of transcript per Million mapped reads) Data publicly available on Flybase http://flybase.org/ [29].

**Fig 2:**
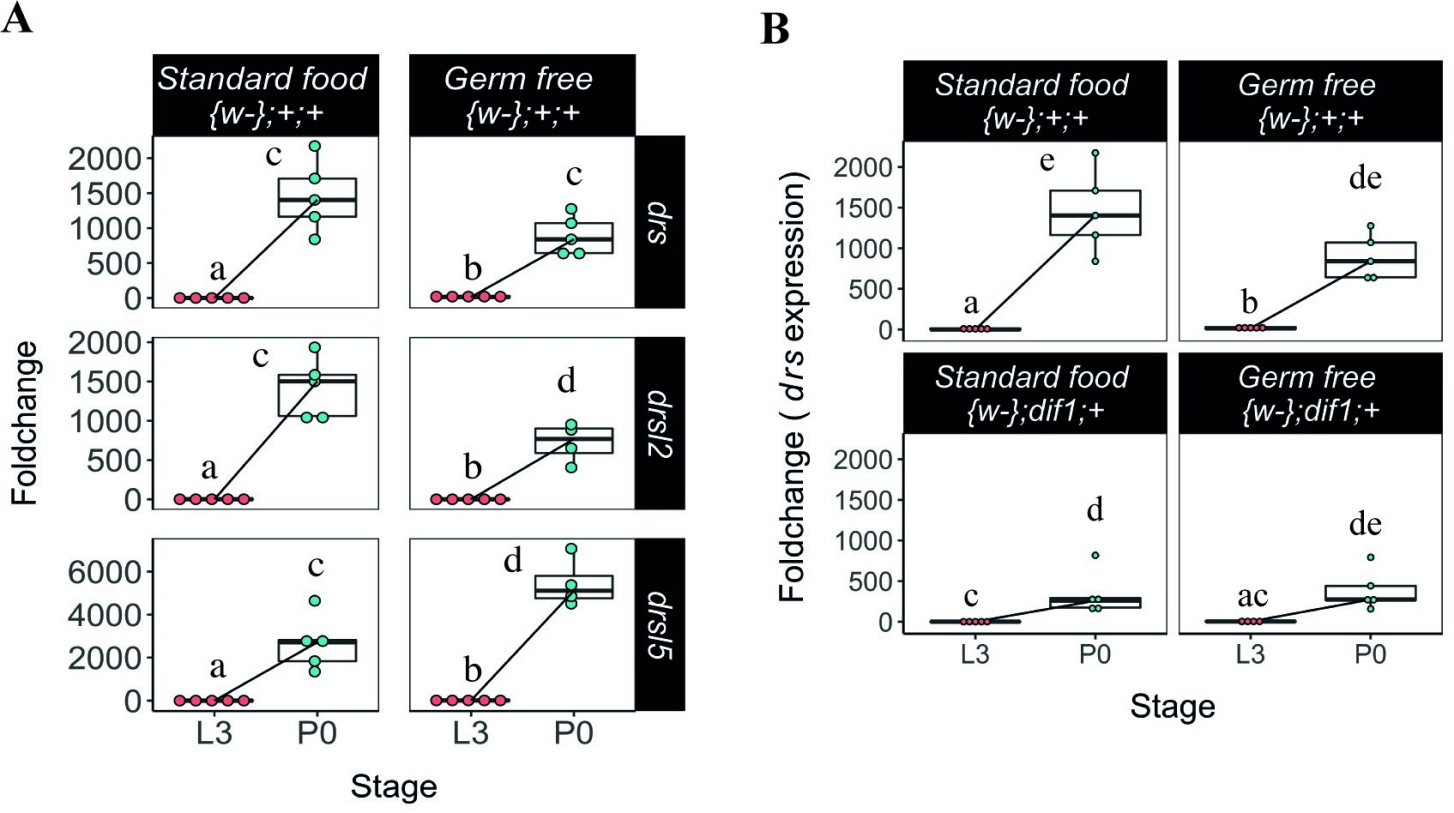
Expression of *drs*, *drsl2* and *drsl5* peaks at pupariation regardless of bacterial presence. **(A)** The expression levels of *drosomycin (drs)*, *drosomycin-like 2 (drsl2)* and *drosomycin-like 5 (drsl5)* increase at pupariation *(drs (lsmean*, pairwise~{w-};+;+ | Stage*drs<0.*0001)*, drsl2 (lsmeans(pairwise~{w};+;+|Stage*drsl2 <0.*0001))* and drsl5 (lsmeans(pairwise~{w-};+;+|Stage*drsl5<0.*0001))* and are not lost under GF conditions (lsmeans(pairwise~{w};+;+|Stage*drs <0.0001); lsmeans(pairwise~{w-}; +;+|Stage*drsl2<0.0001); lsmeans(pairwise~{w};+;+|Stage*drsl5 <0.0001)) (see also S2 Table and S1 Fig). L3 refers to larvae 24 hours after L3 moult and P0 are white pre-pupae (motionless white pre-pupae with evaginated anterior spiracles). Foldchange was determined using the ΔΔCT method. Different letters represent statistically significant differences in foldchange. **(B)** *drs* expression at pupariation is not dependent on the expression of the NF-κB factor, *dif*, both in standard—*(lsmeans*, *pairwise~Stage | +;+;+)<0*.*0001)*, and germ free—*(lsmeans*, *pairwise~Stage | +;+;+)<0*.*0001)* (See also S3 Table). Each dot represents a sample of five pooled individuals; the lines connect the median of the samples at L3 and P0; different letters represent statistically significant differences in fold-change. Fold-change was determined using the ΔΔCT method.

As previously mentioned, a strong peak of AMP expression can be observed at metamorphosis entry (Fig 1) [29]. Around 95% of the anti-microbial peptide (AMP) peak at pupariation, as represented by the white pre-pupal stage (P0), consists of three AMPs: *drs*, *drsl2* and *drsl5* (Fig 1). Another 16 AMPs have been quantified at this stage and show no expression or, comparatively, incipient levels two to three orders of magnitude below *drs*, *drsl2* and *drsl5* (Fig 1 and S1 Table). The strong upregulation of these AMPs could be caused by an ineffective gut purge upon gut remodelling at metamorphosis, which would lead to the canonical activation of immunity pathways. To test if the presence of bacteria constitutes the trigger for the upregulation of *drs*, *drsl2* and *drsl5*, we performed real-time quantitative PCR (qPCR) in mid-third instar (L3) larvae (24 hours after L2-L3 moult; L3 hereafter) and newly pupariated individuals (motionless white pre-pupae with evaginated anterior spiracles; P0 hereafter) raised on germ-free (GF) or standard food conditions. We confirmed an increment in expression at pupariation for *drs*, *drsl2* and *drsl5*, which is not lost under germ-free (GF) conditions revealing its independence from the presence of microbes, including yeast (Fig 2A and S2 Table). The same is observed for other AMPs such as *metchnikowin* and, to a lesser degree, *drsl3*, whereas *defensin* and *cecropinA1* display an incipient expression increase between L3 and P0 under germ-free (GF) conditions (S1 Fig and S2 Table).

Furthermore, *drs* expression still increases in mutant flies for *dif*, the NF-κB factor that triggers *drs* expression upon activation of the Toll pathway by bacteria and fungi (Fig 2B and S3 Table). This indicates that *Dif* is required for the expression of *drs* through a bacteria-independent process, which is further confirmed by the fact that *dif* mutants induce *drs* to comparable levels with or without bacteria presence (Fig 2B and S3 Table). Overall, expression of *drs* at pupariation is mostly independent of bacterial signalling but still partially dependent of the action of the NF-κB factor, *dif*. We also examined for a putative role in this process of *dorsal* (*dl*), the other NF-κB factor that responds to Toll signalling [32–34]. Although this transcription factor appears to have an incipient role in adult *D*. *melanogaster* immunity compared to *dif* [33], the few data regarding other life stages prompted us to test the influence of *dl* on *drs* expression [34]. The expression levels of *drs* in heterozygous individuals for the *dl1* allele show a raise between L3 and P0 that is not different from the one observed in wild-type individuals (S2A Fig). However, given the potential haplosufficiency of this allelic combination, the observed tendency to reduce that peak asks for future clarification.

Together, these results reject the hypothesis that expression of the three main AMPs at pupariation is dependent on microbial induction and suggest that it may be hardwired to the developmental programme.

### Ecdysone regulates the expression of *drs*

**Fig 3:**
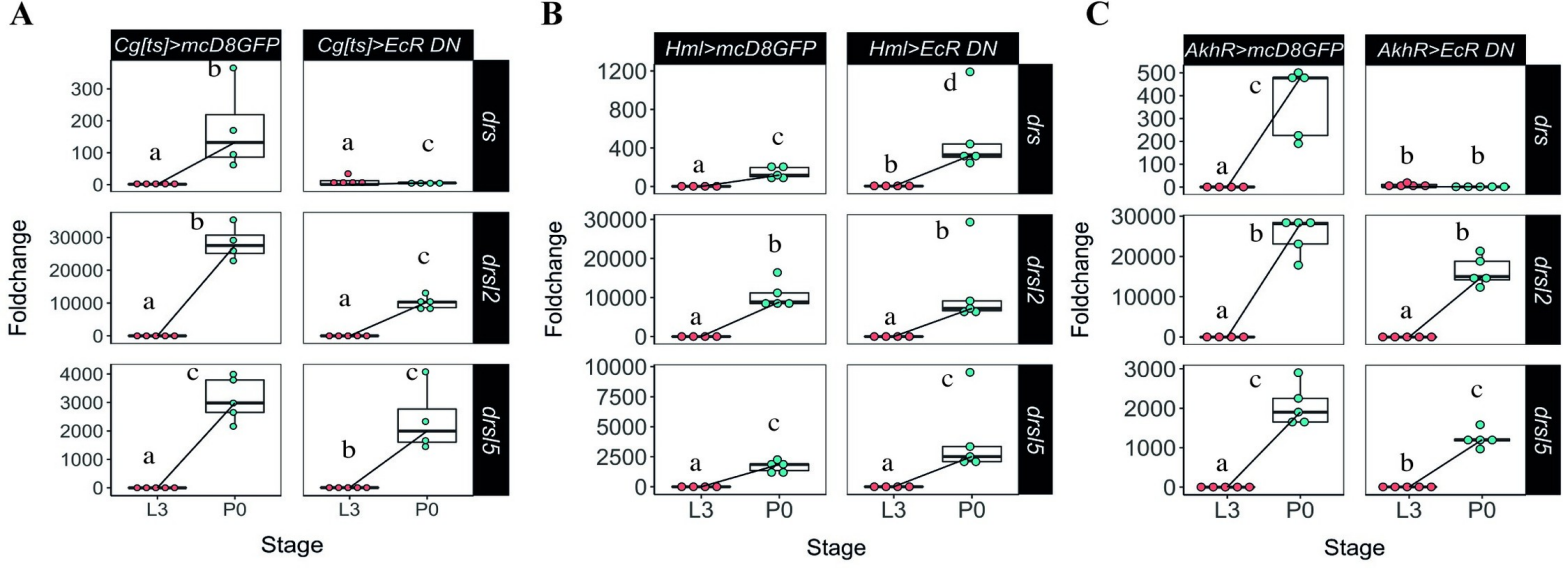
Expression of *drs* at pupariation is developmentally regulated by ecdysone in the fat body. **(A)** Foldchange increase in drs expression (but not of *drsl2* or *drsl5*) at pupariation was eliminated when ecdysone sensitivity is decreased specifically in the fat body and haemocytes simultaneously (*lsmeans(pairwise~Stage|Cg[ts]>mcD8GFP)<0*.*0001*, *lsmeans(pairwise~Stage|Cg[ts] >EcRDN) = 0*.*0017*, *lsmeans(pairwise~Genotype|P0) <0*.*0001)*) (See also S4 Table). **(B)** Reduced ecdysone sensitivity in the haemocytes does not affect the expression of the AMPs under study (*lsmeans(pairwise~Stage|Hml> mcD8GFP)<0*.*0001*, *lsmeans(pairwise~Stage|Hml>EcR DN)<0*.*0001*) (See also S5 Table). **(C)** Reduced ecdysone signalling in the fat body resulted in decreased *drs* expression at pupariation (lsmeans(pairwise~Stage|AkhR> mcD8GFP <0.0001, lsmeans(pairwise~Stage|AkhR>EcRDN) = 0.6709), without affecting the expression of *drsl2* and *drsl5* (lsmeans (pairwise~Stage|AkhR>mcD8GFP)<0.0001, lsmeans(pairwise~Stage|AkhR> EcRDN) <0.0001), for both genes) (See also S6 Table). L3 refers to larvae 24 hours after L3 moult and P0 are white pre-pupae (motionless white pre-pupae with evaginated anterior spiracles). Foldchange was determined using the ΔΔCT method. Different letters represent statistically significant differences in foldchange.

We focused primarily on two main sources of AMP expression with systemic action, the fat body and haemocytes [29]. We manipulated ecdysone signalling sensitivity in these tissues, simultaneously and specifically, using the *Cg-Gal4* driver. We combined *Cg-Gal4* with *tub-Gal80[ts]*, a thermo-sensitive allele of *Gal80* under the control of the tubulin promoter (hereafter *Cg[ts]*), ensuring no perturbation of normal development and pupariation timing, to drive the expression of a dominant negative ecdysone receptor under the control of the UAS sequence [17] *(EcR-A DN W650A TP3*, hereafter *EcRDN*). The reduction of ecdysone sensitivity was triggered from the onset of L3 or at wandering stage, and resulted in the elimination of the *drs* peak at P0, compared to control conditions (Fig 3A). However, the expression increase of *drsl2* and *drsl5* (Fig 3A and S4 Table) and of other AMPs such as *defensin*, *diptericin* and *drsl3* was not affected upon ecdysone signalling reduction in the fat body and haemocytes (S2B Fig and S4 Table). Interestingly, in these conditions two of the AMP genes containing putative EcR binding sites [30], *metchnikowin* and *cecropinA1*, display a suggestive, but statistically not significant, reduction in the wild-type expression increment between L3 and P0.

This result was further confirmed by targeting haemocytes and fat body independently, using the specific drivers *Hemolectin*-Gal4 (*HmlΔ-Gal4*) for the haemocytes and *Adipokinetic hormone receptor*-Gal4 (*AkhR-Gal4)* for the fat body. When ecdysone sensitivity was reduced in the haemocytes, expression of *drs*, *drsl2* and *drsl5*, as well as of other four AMPs, was unaffected (Figs 3B and S2C, and S5 Table). However, although reduced ecdysone sensitivity in the fat body (*AkhR>EcRDN*) did not affect the expression of *drsl2* and *drsl5* (Fig 3C) or of other AMPs (S2D Fig and S6 Table), it resulted in decreased expression of *drs* at pupariation (Fig 2C). It is noteworthy to mention that this reduction of *drs* expression is not likely due to the EcR-mediated disruption of fat body metabolism or abnormal developmental effects because most of the AMP gene expression was not altered.

Altogether, these results indicate that *drs* expression—but not *drsl2* or *drsl5—*is systemically regulated by ecdysone signalling specifically in the fat body at pupariation.

### Expression of *drsl2* in the midgut is regulated by ecdysone

**Fig 4:**
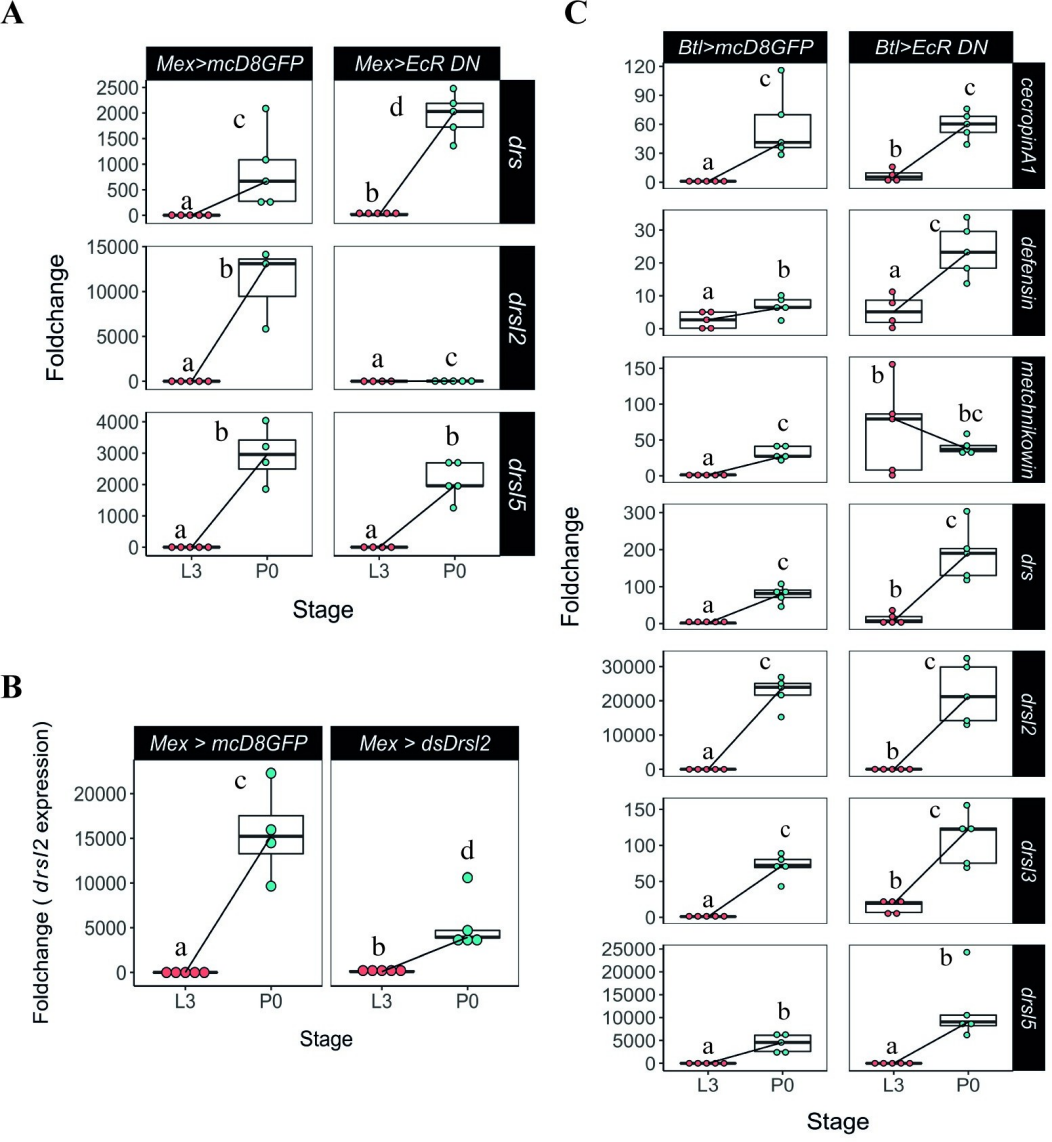
Gut and tracheal expression of relevant AMPs. **(A)** Manipulation of ecdysone sensitivity specifically in the midgut reduced the expression of *drsl2* (*lsmeans(pairwise~Genotype|P0) <0*.*0001; lsmeans(pairwise~Stage|Mex> EcRDN) <0*.*0001*)), but not of *drs* (*lsmeans(pairwise~Stage|Mex>EcRDN)<0*.*0001*) or *drsl5* ((*lsmeans (pairwise~Genotype|P0)<0*.*4479; lsmeans(pairwise~Stage|Mex>EcRDN)<0*.*0001*) (See also S7 Table). **(B)** Knock-down of *drsl2* specifically in the midgut resulted in an overall decrease in the expression of this AMP (*lsmeans (pairwise~Genotype|P0)<0*.*0237; lsmeans(pairwise~Stage|Mex>ds-drsl2) <0*.*0001*). Each dot represents a sample of five pooled individuals; the lines connect the median of the samples at L3 and P0. **(C)** Ecdysone signalling in the trachea does not affect the expression of any AMP under study (*lsmeans(pairwise~Stage|btl>EcRDN)<0*.*0001* for all AMPs, except *metchnikowin* (*pairwise~Genotype|P0)<0*.*6789; lsmeans(pairwise~Stage|btl>EcRDN) = 0*.*3711))*. (See also S8 Table). Foldchange was determined using the ΔΔCT method. Different letters represent statistically significant differences in foldchange.

Having determined that the expression of *drsl2* and *drsl5* is developmentally regulated but does not involve the fat body, we turned our attention to two of the most important tissues for local immune response: the epithelial layers of gut and trachea [35]. First, we overexpressed *EcRDN* specifically in the most abundant cells of the midgut, the enterocytes, using the specific driver *Mex-Gal4* (*Mex*>*EcRDN*). Unlike the fat body, the gut is not responding to the pupariation ecdysone pulse by increasing *drs* expression (Fig 4A). In contrast, decreased ecdysone sensitivity specifically in the midgut resulted in *drsl2* expression levels very close to the L3 basal levels, a drastic reduction (*circa* 600-fold) compared to control P0 levels (Fig 4A). Interestingly, we failed again to detect any difference in the expression of *drsl5*, one of the three main players in the AMP ecdysone-dependent peak (Fig 4A and S7 Table). However, other tested AMPs also appear to be, at least partially, regulated in the midgut at pupariation since, under ecdysone-signalling down-regulated conditions, significant decreases in mRNA levels were observed (S3 Fig).

To further test that the gut is the main source of *drsl2* expression, we knocked down *drsl2* specifically in the midgut through RNAi (by expressing double stranded RNA for *drsl2* under UAS control) (*Mex>ds-drsl2*). This resulted in an overall decrease in the expression of this AMP at pupariation, confirming that the midgut is the primary source of the ecdysone-dependent production of *drsl2* at this stage (Fig 4B).

On the other hand, decreased ecdysone sensitivity in the trachea, using the specific driver *btl-*Gal4 (*btl>EcRDN*), did not have an impact on the expression of any of the three major AMPs under study (Fig 4C and S8 Table). These results show that a layer of local AMP action is at play through the sensing of the ecdysone peak at the onset of pupariation in the gut epithelium.

Together the local and systemic mechanisms of action that share ecdysone control, consisting of *drsl2* and *drs*, account for about 70% of the pupariation AMP peak. Contrastingly, the data also point towards an alternative mechanism for regulating the expression of *drsl5* at pupariation, which is either independent of ecdysone or is regulated by this hormone in an alternative tissue or cell type that were not tested.

### Br is involved in systemic *drs* induction through the fat body at pupariation

Next, we focused our efforts in trying to uncover more of the molecular regulation of *drs* expression, the systemic arm of this ecdysone co-opted immune action.

**Fig 5:**
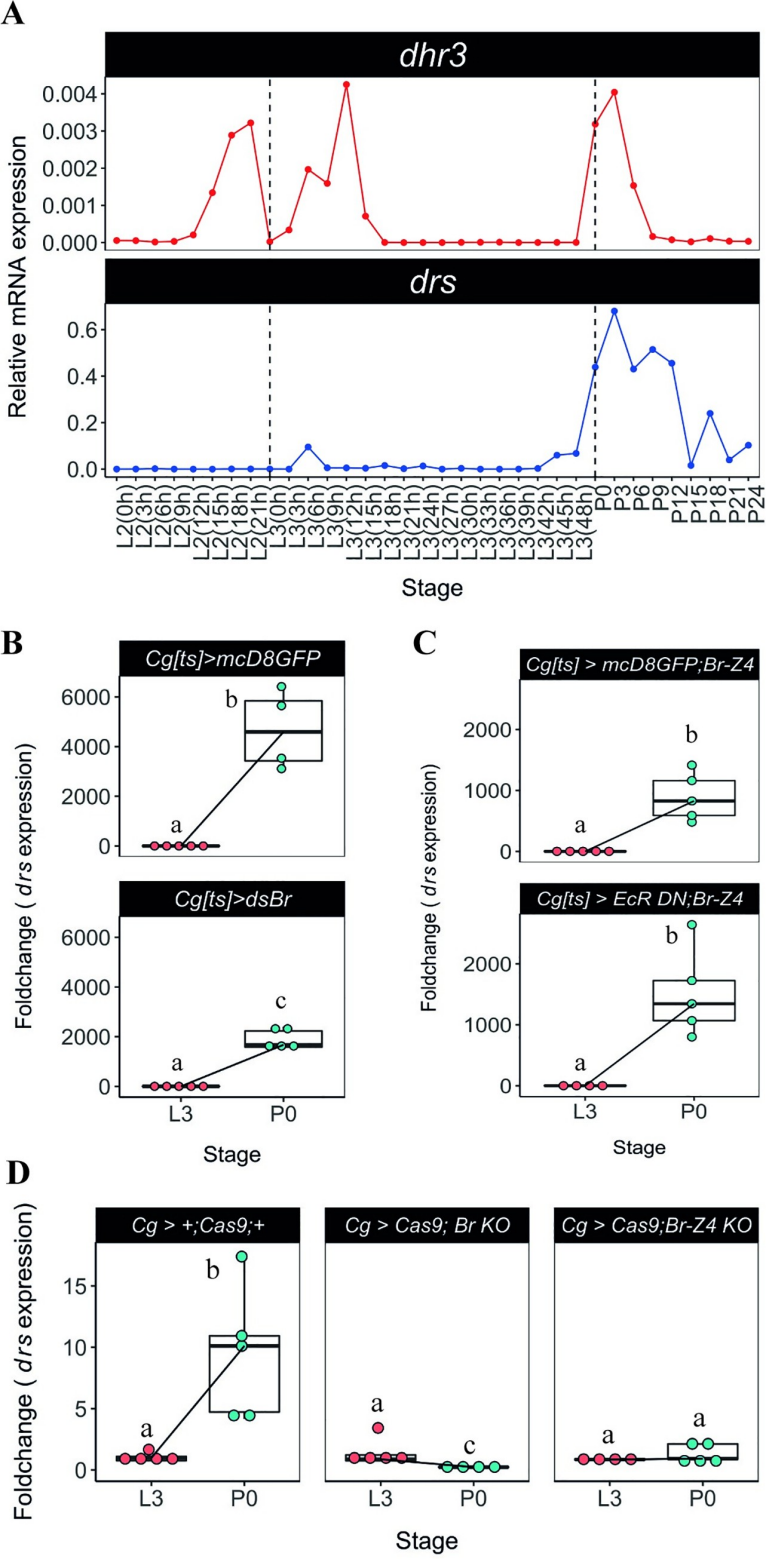
Ecdysone signalling regulates fat body expression of *drs* at pupariation via *Br-Z4*. **(A)** *drs* expression is temporally correlated with a proxy of ecdysone activity, *dhr3*. Samples of *D*. *melanogaster* larvae and pupae were precisely staged at the onset of the instars and collected every 3h. The expression of *dhr3* and *drs* was determined by qPCR for each timepoint. Dashed black lines represent the moult to the L3 or pupariation. **(B)** Knockdown of *br* specifically in the fat body and haemocytes leads to decreased expression of *drs* at P0 (*lsmeans(pairwise~Genotype|P0) = 0*.*0510*). (**C**) Co-overexpression of *br*-*Z4* when ecdysone sensitivity is decreased in the fat body and haemocytes is sufficient to rescue the expression of *drs* at pupariation (*lsmeans(pairwise~Genotype|P0) = 0*.*3068)*. (**D**) Conditional CRISRP-mediated knock-outs for the common region of *br* and *br-Z4* lead to loss of *drs* expression at pupariation (*lsmeans(pairwise~Stage|Cg> +;Cas9;+) <0*.*0001;lsmeans(pairwise~Stage| Cg>Cas9;br KO) = 0*.*0001*, *lsmeans(pairwise~Stage|Cg>Cas9;br-Z4 KO) = 0*.*4691)*. Each dot represents a sample of five pooled individuals; the lines connect the median of the samples at L3 and P0; different letters represent statistically significant differences in foldchange, determined using the ΔΔCT method.

Previous RNAseq data showed that the expression of *drs* markedly increases at pupariation and gradually drops the expression in next 24 hours (Fig 1, [36]). We further confirmed that the expression of *dhr3* (an early-late gene of the ecdysone signalling cascade directly regulated by the ecdysone-ecdysone receptor complex) and that of *drs* are tightly correlated and peak exclusively at pupariation (Fig 5A). Importantly, this robust expression of *drs* at the onset of metamorphosis seems to be a general feature regardless of their genetic background (S4 Fig).

*br* is an early gene of the ecdysone signalling cascade and a direct target of the ecdysone receptor, characterized as a holometabolan pupal specifier with increased expression before pupariation [31,37,38]. Because the transcriptional peak of *drs* is only found at the moult from the L3 to pupa but not from the L2 to L3, Br seems a good candidate for regulation of the *drs* expression. We thus examined the association between *br* and the ecdysone-sensitive systemic induction of *drs* at metamorphosis by knocking-down *br* specifically in the fat body and haemocytes. This led to a significant decrease in the expression of *drs* at pupariation, when compared to the control (Fig 5B), suggesting a role for *br* in the regulation of *drs* expression. To further explore this possibility, we simultaneously reduced ecdysone signalling and restored *br* in the haemocytes and fat body. This was sufficient to rescue the expression of *drs* to control levels at P0 (Fig 5C). Furthermore, we generated two different conditional CRISPR-mediated knock-out constructs [39,40], one targeting a common region to all *br* isoforms and, the other, a region specific to *br-Z4*, previously associated with AMP expression using *in vitro* techniques in a different species [41]. Knock-out of all *br* isoforms or of *br-Z4* alone in haemocytes and fat body, led to the elimination of the *drs* peak at pupariation (Fig 5D and S9 Table), supporting that Br is involved in the regulation of *drs* expression downstream of the ecdysone receptor complex in the fat body. In contrast, this was not the case for any of the other AMPs considered, particularly *drsl2* and *drsl5*, which show no alteration in expression under these conditions (S5 Fig).

To further explore this regulation of *drs* expression by Br-Z4, we used the JASPAR (http://jaspar.genereg.net) matrix MA0013.1 to run a comprehensive search for Br-Z4 binding sites over 2 kb of putative promoter region that is upstream of the coding sequence of *drs*, obtained from Flybase (http://flybase.net/), using FIMO software (S6A Fig) [42]. We identified three putative Br-Z4 binding sites (p-value<0.0001) in the predicted 5’ promoter region of *drs* (S6B Fig).

Together, these results support the idea that isoform Br-Z4, albeit other isoforms cannot be ruled out, is involved in the regulation of the expression of *drs* either directly or indirectly at pupariation.

### *drs* is necessary to reduce bacteria derived from imperfect larval gut purge

Having establish the basis of the regulation of *drs* at the onset of metamorphosis, we set to determine its relevance to the control of bacterial numbers throughout pupal development. In laboratory *D*. *melanogaster*, the overwhelming majority -between 85% and 99% of all identified bacteria- of the gut microbiota is constituted by the Acetobacter and Lactobacillus genera [43,44]. Before pupariation and metamorphosis, larvae purge their gut contents and any imperfection in this process creates the conditions in the pupa for a putatively rampant infection. Thus, we examined whether or not gut purge eliminates all bacteria from the gut and how this process may be dependent on the systemic action of *drs*.

**Fig 6:**
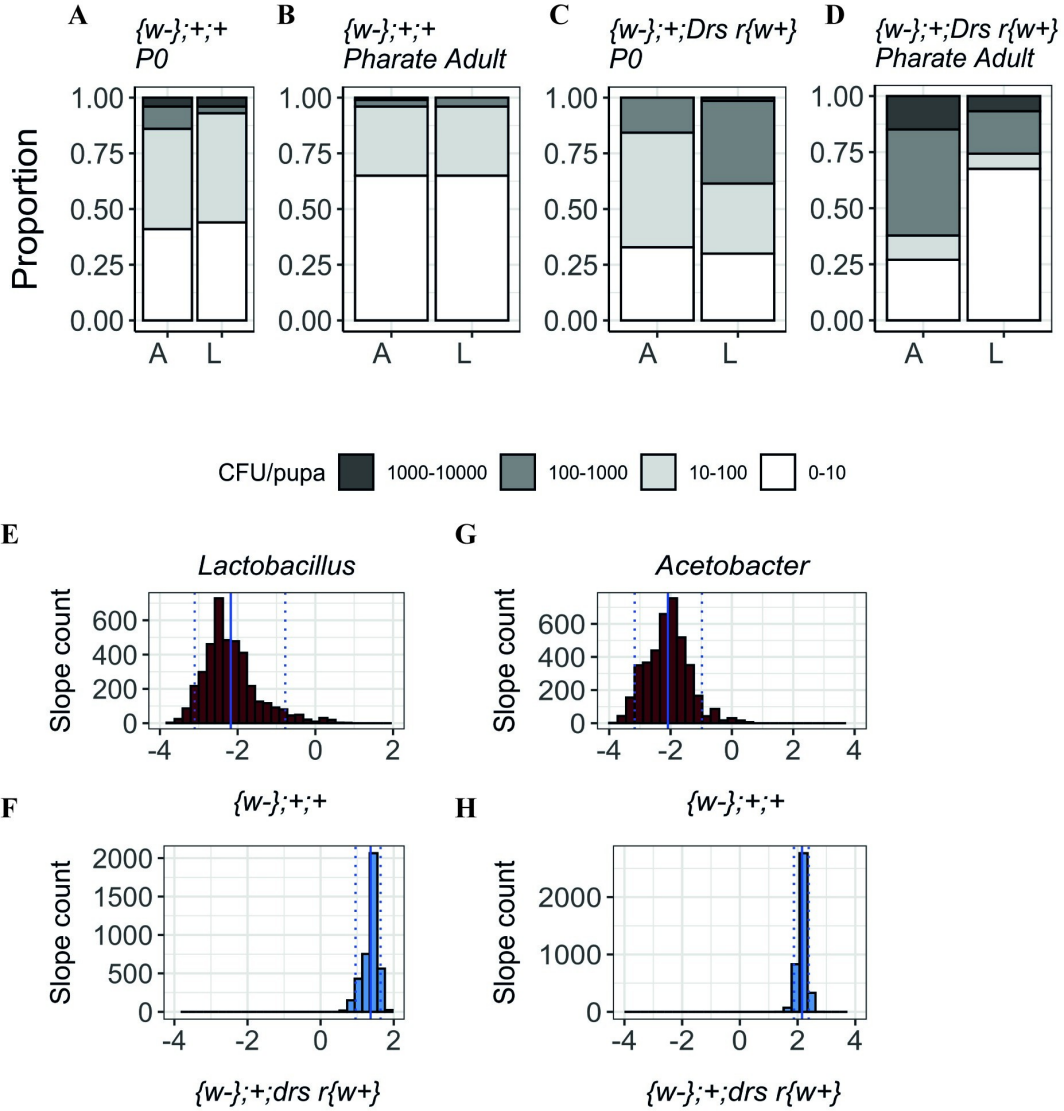
Bacteria quantification (*Lactobacillus* (L) and *Acetobacter* (A)) in P0 pupae and pharate adults in wild-type and *drs* mutants. **(A-B)** In control flies *({w-}*;+;+), the number of pupae with more bacteria decreases between P0 and pharate adult stages. **(C-D)** In the *drs* mutant line *({w-}*;+; *Drs* r{w+}), there are more individuals with higher loads of bacteria persisting from P0 to pharate adult. **(E-F)** Slopes for the number of *Lactobacilli* in control and the mutant lines. **(E)** Control samples have negative slopes indicating a decrease in the number of bacteria between stages (*Median*: *-2*.*308 (IQR*: *-2*.*619; -1*.*880*); One-way Wilcoxon test for median0: <2.2e-16, r = 0.865). **(F)** *drs* mutant samples have positive slope values, representative of an increase in the number of bacteria across metamorphosis (*Median*: *1*.*408 (IQR*: *1*.*286; 1*.*498); One-way Wilcoxon test for median>0*: *<2*.*2e-16*, *r = 0*.*866*). **(G)** Control samples distribution show negative slopes, i.e., a decrease in the number of bacteria between stages (*Median*: *-2*.*0*.*757 (IQR*: *-2*.*5721; -1*.*7128); One-way Wilcoxon test for median<0*: *<2*.*2e-16*, *r = 0*.*866*). **(H)** *drs* null mutant displays positive slope values (*Median*: *2*.*165 (IQR*: *2*.*077; 2*.*247); One-way Wilcoxon test for median> 0*: *<2*.*2e-16*, *r = 0*.*866*). Doted lines represent the 0.05 and 0.95 quantiles; full line represents the mean slope.

Under our standard rearing conditions, the mortality between P0 and pharate adult is negligible, which allows us to infer and correlate the bacterial numbers detected in each of these developmental stages (S7A Fig and S10 Table). After gut purge, approximately 5% of control P0 individuals contained bacteria in the order of the thousands and another 12% in the order of the hundreds (Figs 6A and S7B). However, at the pharate adult stage, the number of individuals in these two categories decreased alongside an increase in the number of individuals without detectable bacteria (Figs 6B and S7B). In contrast, *drs* null mutants showed an increase rather than a decrease in the number of pupae with more bacteria between these two stages (Figs 6C, 6D and S7B), supporting that *drs* is necessary to eliminate gut derived bacteria during metamorphosis.

To compare the pattern of variation between P0 and pharate adults across genotypes, we applied a resampling strategy [45]. To this aim, we randomly resampled our experimental data, without replacement, generating a smaller, replicated dataset. For the negative binomial component, positive coefficients mean higher counts for bacteria, while negative mean reduced counts. We find that most coefficients associated with the control line are negative (Fig 6E and 6G). On the other hand, the coefficients associated with *drs* null mutant were mostly positive for both bacteria types (Fig 6F and 6H). These results suggest that, throughout metamorphosis, a process that is dependent on the increase of AMP expression at pupariation is necessary for controlling persisting bacteria originating from gut purge imperfections.

**Fig 7:**
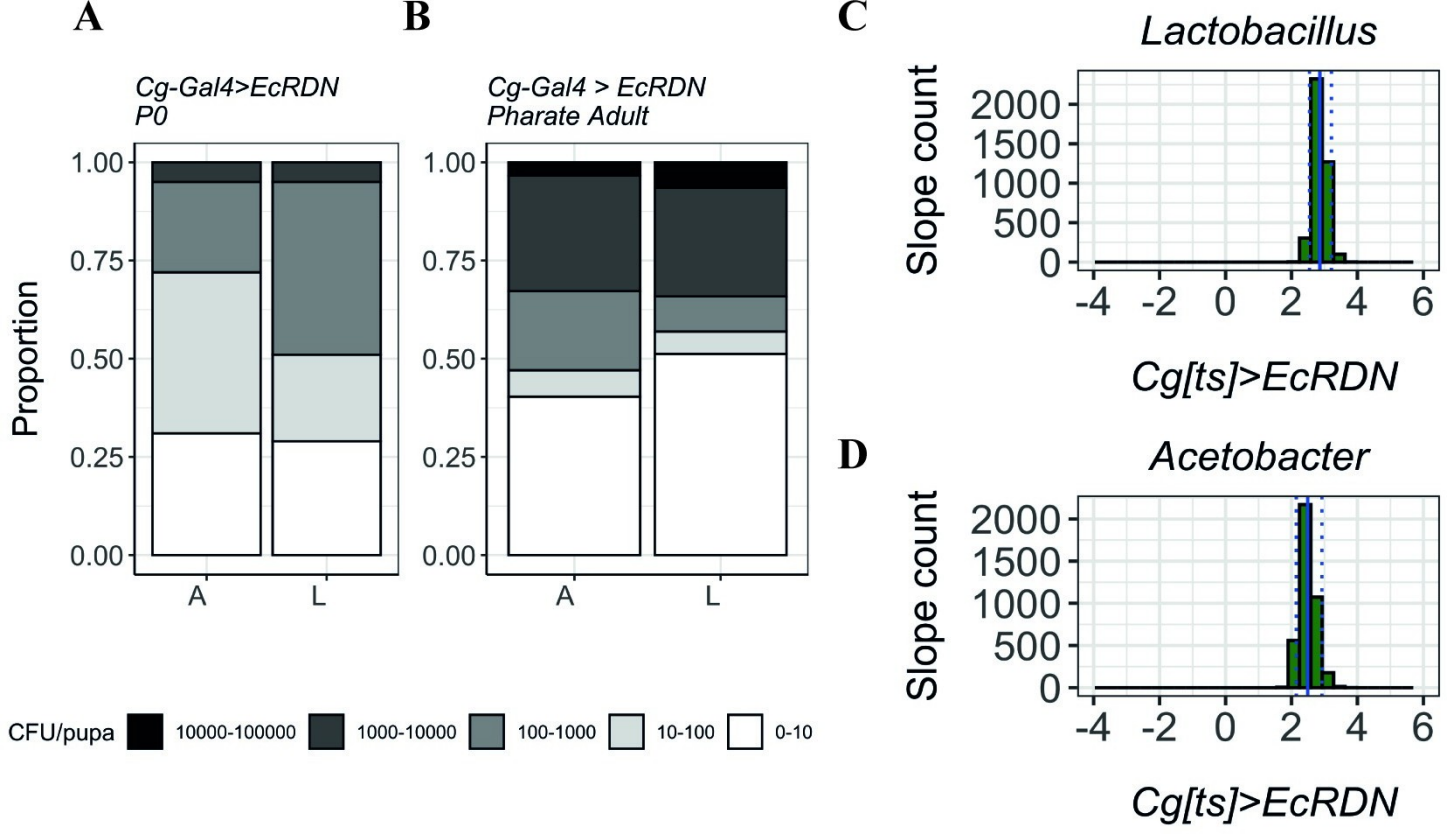
Quantification of Lactobacillus (L) and Acetobacter (A) in P0 pupae and pharate adults without ecdysone signalling in the fat body and haemocytes. **(A-B)** When ecdysone sensitivity is decreased in fat body and haemocytes, for both bacteria types, the number of individuals with increased bacterial loads is significantly higher in pharate adults compared to L3 larvae, revealing a deficit in bacterial growth control. Y-axis represents the proportion of individuals in each bacterial count category. **(C-D)** Coefficient (slopes) distribution in *Cg[ts]>EcRDN* samples of *Lactobacillus* **(C)** (*Median*: *2*.*855 (IQR*: *2*.*724; 2*.*996); One-way Wilcoxon test for median>0*: *<2*.*2e-16*, *r = 0*.*866)* and *Acetobacter* **(D)** (*Median*: *2*.*449 (IQR*: *2*.*308; 2*.*623); One-way Wilcoxon test for median>0*: *<2*.*2e-16*, *r = 0*.*866)*. Doted lines represent the 0.05 and 0.95 quantiles; full line represents the mean slope.

When ecdysone signalling activity is decreased in the fat body and haemocytes (*Cg[ts]>EcRDN)*, a similar pattern can be observed: there is a higher proportion of individuals with larger loads of persisting bacteria (Figs 7A,7B and S7C). Alongside an increase in the number of individuals that resolve the infection (0–10 class), the distribution assumes a much stronger bimodal distribution with more than the triple of individuals in the classes above 10^3 CFUs, and a concomitant shrinking of the intermediate classes (10 to 1000 CFUs). This suggested impaired bacterial clearance during metamorphosis is further confirmed by our resampling strategy-based analysis whereby *Cg[ts]>EcRDN* samples have positive coefficient values for both *Lactobacillus* (Fig 7C) and *Acetobacter* (Fig 7D*)*.

### The local gut *drsl2*-mediated response has a negligible effect in controlling bacterial loads across metamorphosis

**Fig 8:**
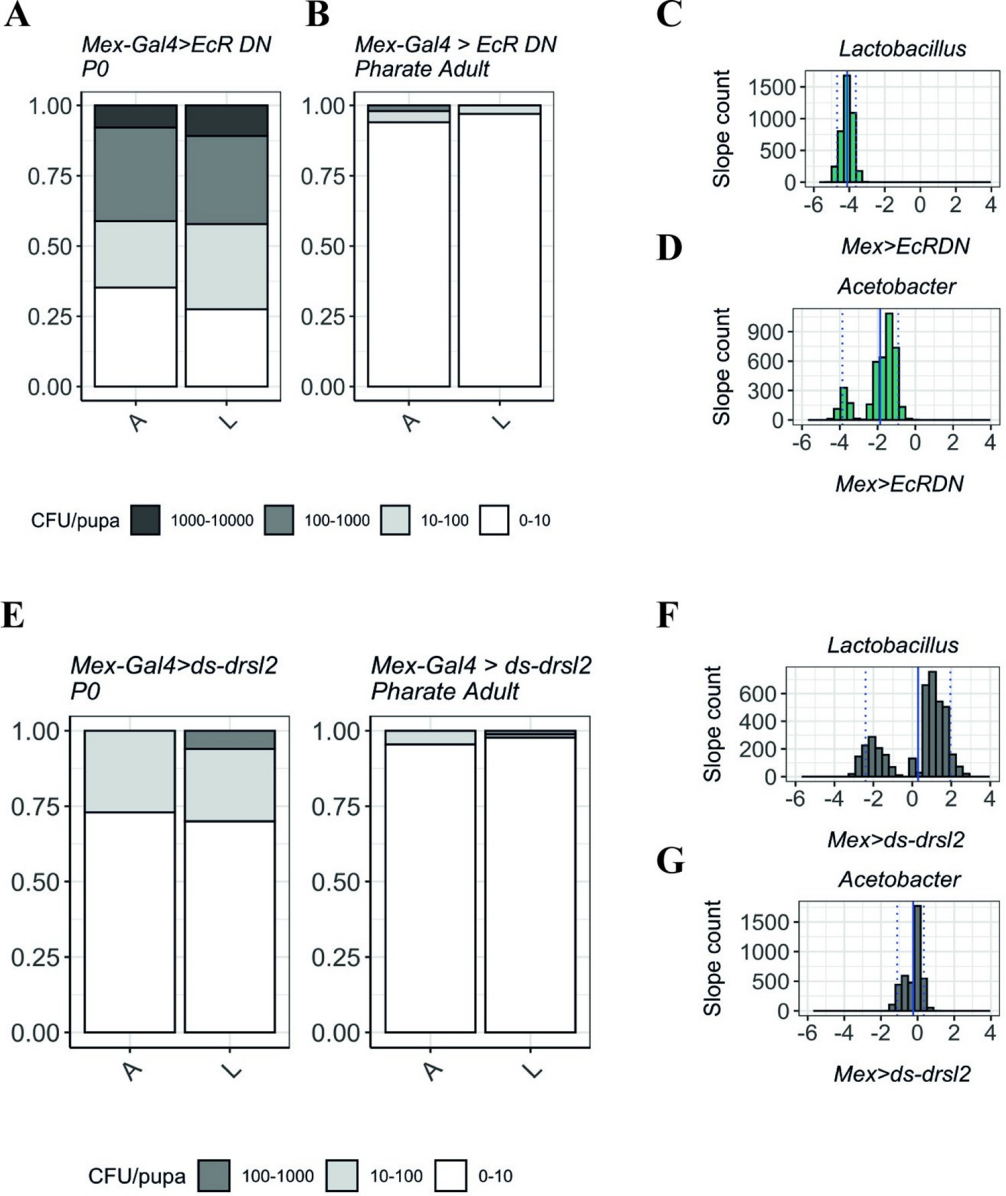
The role of local gut response in controlling the loads of *Lactobacillus* (L) and *Acetobacter* (A) in P0 pupae and pharate adults. **(A-B)** When ecdysone signalling is decreased in the enterocytes, the number of bacteria persisting metamorphosis does not increase. Y-axis represents the proportion of individuals in each bacterial count category. **(C-D)** Decrease in bacteria numbers in *Mex>EcRDN* individuals is reflected by negative coefficient values for both *Lactobacillus* **(C)** (*Median*: *-4*.*091 (IQR*: *-4*.*303; -4*.*108); One-way Wilcoxon test for median<0*: *<2*.*2e-16*, *r = 0*.*866*) and *Acetobacter* **(D)** (*Median*: *-1*.*5413 (IQR*: *-2*.*0930; -1*.*2336); One-way Wilcoxon test for median<0*: *<2*.*2e-16*, *r = 0*.*866)*. Doted lines represent the 0.05 and 0.95 quantiles; full line represents the mean slope. **(E-F)** Upon induction of RNAi against *drsl2* in the midgut (*Mex>ds-drsl2*), we observe a clearance of bacteria between P0 and pharate adult stages indicating a negligible role for *drsl2* in the process. **(G-H)** *Mex>ds-drsl2* samples coefficient not significantly different from 0 (same number of bacteria between P0 and pharate) for *Lactobacillus* (*Median*: *0*.*9044 (IQR*: *-1*.*2893; 1*.*3397); One-way Wilcoxon test for median = 0*: *<1*.*239e-15*, *r = 0*.*126*) and *Acetobacter* (*Median*: *-0*.*09671 (IQR*: *-0*.*64948; -0*.*07480); One-way Wilcoxon test for median<0*: *<2*.*2e-16*, *r = 0*.*399*).

Having established the necessity of *drs* systemic action to control bacterial proliferation in the pupa, we quantified bacterial load progression between L3 and P0 when ecdysone signalling or *drsl2* expression are perturbed in the gut (Fig 8). Disrupting ecdysone signalling in the gut (*Mex>EcRDN*) has no impact on bacterial clearance as considerably higher numbers of individuals are found in low bacterial load classes upon progression from L3 to P0 (Figs 8A,8D and S8A). The same pattern is observed if we induce RNAi against *drsl2* specifically in the gut (*Mex>ds-drsl2)* (Figs 8E, 8H and S8B).

These results suggest that the local immune response does not have a measurable antibacterial effect compared to the (sufficient) systemic response that is still operating in these conditions.

## Discussion

In this study in *D*. *melanogaster*, we establish the association between ecdysone and the increased expression of *drs* and its paralogues (*drsl1-6*) at pupariation and the biological consequence of this process. We determine the functional link between the endocrine and immune systems, acting both systemically and locally during metamorphosis to control the number of bacteria in the pupal case. The biological significance of the AMP peak at the onset of metamorphosis is revealed by the systemic expression of *drs*, as both mutant and ecdysone sensitivity-reduced individuals showed an increase rather than a decrease in the number of bacteria between P0 and pharate adult stages. Interestingly the effect was detected for both Gram-positive (*Lactobacillus*) and Gram-negative (*Acetobacter*) bacteria. Although *drs* is generally characterized as an antifungal AMP, previous studies already demonstrated that it can also be induced by both Gram-positive and Gram-negative bacteria [46–52]. As such, the unexpected higher impact of *drs* absence on *Acetobacter* may be explained by a direct action on Gram-negative bacteria. Indeed, a previous study demonstrated that *drs* is induced in the fat body after a systemic infection with *Erwinia carotovora carotovora 15* (*Ecc15*) [53], which suggests this AMP responds to the presence of Gram-negative bacteria and may be relevant under an infection scenario. Alternatively, the impact seen on Gram-negative bacteria might be due to some compensatory mechanism driven by other AMPs in the absence of *drs*. Also of note is the fact that the humoral arm of defence is not alone acting at this stage of development. Regan and colleagues demonstrated that at pupariation, ecdysone is required to activate haemocytes and to orchestrate the active migration of these cells towards wounding sites [7]. Therefore, at this stage both ecdysone-regulated cellular and humoral mechanisms are likely to act in synergy to decrease the probability of a damaging infection.

Surprisingly two of the actors in this process, *drsl2* and *drsl5*, have not been found to have any antibacterial activity [53]. However, both these AMPs are upregulated in the gut upon *Ecc15* infection, which suggests that they may be relevant during an immune challenge [53] and aligns with our findings. Further studies should ascertain the exact mode of action of these drosomycin-like peptides.

Because *drsl2* and *drsl5* are not proven to act as AMPs and given that the lack of local expression of *drsl2* did not affect bacterial persistence *per se*, we focused our efforts on disentangling more of the molecular regulation of *drs* expression in the fat body. We showed that the expression of *drs* at this stage depends upon the expression in the fat body of the ecdysone receptor target, *br-Z4*. This is in accordance with previous indications that Br is essential for the PGRP-LC-independent hormonal regulation of *drs* in S2 cells [4]. However, these previous studies did not demonstrate this association *in vivo*, thus not establishing the spatial and temporal range of Br action on AMPs nor its relation to pupal viability. Furthermore, similar ecdysone-mediated immune activation through Br has been previously observed. Studies in the silkworm *Bombyx mori* showed that during metamorphosis, ecdysone activates the transcription of lysozyme via Br-Z2 [54] and of the AMP *lebocin* via Br-Z4 [41].

This intermediary role of Br in the regulation mechanism of ecdysone over the AMPs may explain why these effectors are only upregulated at pupariation, as revealed by our expression profile data. Although ecdysone controls all the moults throughout development, *drs* expression increases in response to this hormone specifically at pupariation, when Br is expressed in Holometabola [31,55]. The regulation of AMPs by Br has been implicated in the interpretation of AMP expression throughout development between holo- and hemimetabolan insects. Johnston and colleagues demonstrated that these two insect groups have different AMP expression patterns, with holometabolans showing a strong upregulation of immunity that coincides with metamorphosis timing and Br expression [56]. In contrast, hemimetabolans do not undergo a dramatic increase in immunity, possibly correlating with much less drastic changes in body remodelling during progressive metamorphosis. In accordance, there is indication that holometabolan insects suffer a reduction in density, diversity and alter the composition of bacterial populations during metamorphosis [57–59], whereas hemimetabolans show a constant increase in microbial density and diversity throughout development [60]. These data, as ours, are consistent with the previously proposed hypothesis that complete metamorphosis elicits a prophylactic immune response [21,22]. However, until now it was unclear how ecdysone could temporally regulate the expression of AMPs at pupariation.

Alterations in immunity at life stages with higher risk of infection seem to be a general feature of development, finding some parallels in both vertebrates and invertebrates. In *Drosophila triauraria*, genes of the *drs* family are induced during diapause, which is also considered to be a more susceptible period [61]. Most interestingly, data from the clawed frog, *Xenopus laevis*, also point towards an association between the timing of metamorphosis and a shift in the immune response [62]. For example, Major Histocompatibility Complex I (MHC class I) molecules, which bind to peptide fragments derived from pathogens, are first detected at the beginning of metamorphosis [63] and the expression of MHC class II molecules is not triggered if metamorphosis is chemically blocked [64].

Together, our data suggest a regulatory mechanism through which ecdysone co-opted the immune system possibly to enhance the success of metamorphic processes. This study reveals a novel association between the endocrine and immune systems in *D*. *melanogaster*, which act both systemically and locally during metamorphosis. This recruitment of immunity mechanisms by the holometabolan metamorphosis programme presents other interesting features. Three AMPs, *drs*, *drsl2* and *drsl5*, explain around 95% of the peak observed at the L3 to pupal transition. Interestingly, these three AMPs have been co-opted differently and complementarily to produce what we interpret as a full-fledged immune prophylactic response despite the absence of solid evidence of an antibacterial role for *drsl2* and *drsl5*. However, this speculation is tempting. *Drsl2*, was recruited to one of the main exposed surface epithelia of larvae, the midgut, that undergoes extensive remodelling at metamorphosis and *drs* is produced in the fat body at metamorphosis initiation, exerting its antibacterial action in a systemic fashion. These two processes, with local and systemic action, account for close to 70% of the pupariation AMP peak and share a mechanism of ecdysone control also observed in other anticipatory immune responses [14].

This multi-layered developmentally-regulated immune response at the onset of pupariation adds to the growing notion of in-built anticipatory mechanisms, underscores the sophistication and versatility of the co-option process and hints at a role in the evolutionary success of metamorphosis [65–72].

## Materials and methods

### *Drosophila* stocks and husbandry

If not otherwise noticed, *w[1118]* flies were obtained from Dr. Luís Teixeira (Instituto Gulbenkian de Ciência, Portugal) and used as the reference wild-type strain. The following lines were obtained from the Bloomington Drosophila Stock Center: *w;UAS-mCD8GFP* (#5137), *Cg-Gal4* (#7011), *tub-Gal80[ts]*(#7017), *w; UAS-Cas9 P2*(#58986), *w;UAS-brZ4* (#51193) and *w;UAS-dsBroad* (#104648). RNAi inducing stock, UAS-*ds-drsl2* (v109207) was obtained from the Vienna Drosophila Resource Center. Several stocks were generously shared by our colleagues, Drs Lynn Riddiford, University of Washington, USA (*w;UAS-EcRADN TP3 W650A*), Marc Dionne, Imperial College London, UK (*HmlΔ-Gal4*), François Leulier, IGFL, France (*Mex-Gal4*), Kim Rewitz, University of Copenhagen, Denmark (*AkhR-Gal4*), Bruno Lemaitre, EPFL, Switzerland (*iso;iso;Drs*^*r(w+)*^ and the respective background control). The rescue lines *w;UAS-mCD8GFP; P{UAS-br*.*Z4}37–6* and *w;UAS-EcRADN TP3 W650A; P{UAS-br*.*Z4}37–6* were generated in house at Instituto Gulbenkian de Ciência (Portugal) by the Fly Transgenesis Service (Dr Gastón Guilgur). Standardly raised flies were kept on food with the following composition (g/mL): 4,5% molasse, 7,5% sugar, 7% cornmeal, 2% yeast extract, 1% agar, 2,5% Nipagin 10%. All fly lines we kept at 25°C in a 12L:12D photoperiod. For the ecdysone decreased sensitivity experiment and tissue specific CRISPR, animals were maintained at 17°C until the moult to the L3 and then transferred to 29°C to activate Gal4 activity.

### Larval and pupal staging

Larvae were resynchronized every two hours and kept in normal food for the appropriate time before collection in Trizol. Egg collections were performed on standard *Drosophila* food plates at 25°C, unless mentioned otherwise. All larvae used for this study were resynchronised either at the onset of the L2 or L3, as previously described [73]. Eggs were collected every four hours and larval densities kept around 200 larvae/60 mm diameter plate. Newly moulted L2 or L3 larvae were collected every two hours and raised at 25°C (up to a maximum of 30 larvae/vial) on standard food. From these collections, larvae were collected at 24 hours past L3 moult to establish the L3 qPCR time point used in every experiment on Figs 2 to 5 (qPCR details below). For collection of white pre-pupae (P0), bottles with L3 larvae were monitored every 20 minutes, between 48-hours and 72-hours after the moult to L3. Across this study, P0 were identified as motionless white pre-pupae with evaginated anterior spiracles. After collection, every sample was immediately stored in Trizol and stored at -80°C until RNA extraction.

### RNA extraction, cDNA synthesis and real-time quantitative PCR

RNA was isolated using the Direct-zol RNA MiniPrep Kit (Zymo), following manufacturer’s instructions. DNase I treatment and cDNA synthesis were preformed using the RQ1 RNASE-FREE DNASE 1 (Promega) and the RevertAid H Minus First Strand cDNA Synthesis Kit (Thermo Scientific), respectively. qPCR was performed using the SYBR Green PCR Master Mix (Applied Biosystems) and ABI 7900HT (Applied Biosystems). The PCR conditions used in all experiments were: initial denaturation/ enzyme activation, 95°C for 10’; followed by 45 cycles of denaturation, 95°C for 10”; annealing, 60°C for 10”; extension, 72°C for 30”. Specificity of PCR amplification was verified by melting curve: 95°C for 10”, 65°C for 1’, 97°C for 1”; cooling, 37°C, 30”. Primers used for all qPCR reactions are represented in S11 Table. Foldchange was determined using the ΔΔCT method, which compares the expression of a given gene of interest to a housekeeping gene (*rpl32*) between experimental and control samples [74]. Control L3 samples were used as reference. The data was analysed with a general linear model, using the genotype, stage and gene as explicable variables, as follows: glm(log(Foldchange)~Genotype*Stage*Gene, data = mydata). The expression level for each gene at P0 and pharate was compared within and between genotypes through multiple comparisons using the *lsmeans* package from Rstudio 1.2.5033.

### Germ-free flies

Newly laid eggs were collected into a sterile embryo basket and washed with autoclaved MilliQ water. The eggs were dechorionated and sterilised using a 13% Bleach solution (Sigma Aldrich) for 10 minutes, washed again with Milli-Q water and sterilized using a 1% Virkon solution for 2 minutes. The sterilized embryos were transferred to axenic media with or without antibiotics (200 mg/mL Rifampicin, 100 mg/mL Tetracycline, 100 mg/mL Streptomycin, 15 mg/mL gentamicin). The axenic media contained all the normal ingredients of fly food, is autoclaved prior to use and supplemented with the fungicide Bavistin, which guarantees absence of any traces of microorganisms, including yeast. All plates and bottles were kept inside sterilised containers at 25°C and all manipulations were conducted in a horizontal flood hood, using sterile materials. After the treatment, the germ-free status was validated by plating surface-sterilized adult flies (from both germ free and control conditions) onto MRS and D-Mannitol media.

### P0 and pharate adult collection and plating

P0 samples were collected as aforementioned. Pharate adults were identified as fully developed adults (with tanned wings and pigmented eyes) still to eclose. Pharate adults were dissected out of the pupal case and collected in 20 minutes intervals 48h to 72h after P0 collection. All samples were surface sterilized once with Milli-Q water, 70% ethanol and 13% bleach and rinsed (3x) with sterile water to eliminate bleach traces. After these treatments, each pupa was macerated in 300 μL of LB medium, of which 50 μL were spread onto MRS or D-Mannitol plates. The plates were incubated at 25°C for 48h for bacterial growth. For each stage and medium a sample size of 100 pupae/pharate was collected per genotype.

### Statistical analysis for bacterial growth across stages

To compare the pattern of variation between P0 and pharate adults across genotypes, we followed a resampling strategy [45]: the initial sample was resampled in subsets of 60 entries, without replacement, 4000 times, generating a smaller, pseudo-replicated dataset. In each cycle, a zero-inflated negative binomial regression (*zeroinfl (cfu_pupae ~ Stage | Stage*, *dist = "negbin"*,*)*) was performed to model the change of bacteria according to the stage. This type of regression, does not assume the variance and the mean of the data to be equivalent. In addition, this regression models the zeros independently, using a logistic regression from the count values that considers zero as the event of interest, modelled using a negative binomial regression. After running this regression, we obtain two regression coefficients, one for the binomial component and another for the negative binomial.

At the end of the 4000^th^ cycle, a set of these coefficients was obtained for each genotype. This strategy allows the estimation of a set of coefficients for both the logistic regression (focused on the number of zeros) and the negative binomial (modelling the counts). This set permits location and variation statistics, as well as a more accurate estimate of the parameters of the coefficients sampling distribution.

Subsequently, we assessed if the central tendency of the population is greater or smaller than 0 using one-sided hypothesis tests. In parallel, a measure of the effect size was also calculated. Pseudo-replicated data was generated using the *effsize* package in Rstudio 1.2.5033 and the zero-inflated negative binomial model was analysed with the *psc1* package. All graphics were generated using the *ggplot2*, *ggpubr* and *gridExtra* packages.

### CRISPR knock-out flies

The tissue-specific UAS *br* CRISPR knockout constructs were cloned into pCFD6 vector (www.crisprflydesign.org, Addgene #73915) according to manufacturer’s instructions. Four independent gRNA constructs were cloned to target introns (see primers listed in S12 Table) of the *br* core region genomic DNA sequence for *br* common construct and Z4 isoform specific sequence for *br-Z4* construct to target only native genes but not transgenes. After being sequenced, the constructs were inserted on either the second chromosome in a recipient line carrying phiC31 integrase and attP40 landing site with an in-house modified line (*y*,*w*, *P{y+*.*nos-int*.*NLS}; P{CaryP y+}attP40*) or the third chromosome in a recipient line carrying phiC31 integrase and attP2 landing site, *y*,*w*, *P(y[+]*.*nos-int*. *NLS); P(CaryP)attP2* (gift from Dr. Diogo Manoel, Sidra Medical and Research Center, Qatar). Transgenesis was carried out in-house at Instituto Gulbenkian de Ciência (Portugal) by the Fly Transgenesis Service (Dr Gastón Guilgur).

### Br binding sites prediction

The DNA binding motif matrix for Br-Z4 was obtained from the JASPAR database (http://jaspar.genereg.net) and the 5’ 2kb region of the drosomycin gene was acquired in Flybase (http://flybase.org). Both sequences were uploaded in FIMO software (http://meme-suite.org/tools/fimo) [42], using a p-value < 0.0001 as filter.

## Supporting information

S1 Fig The expression levels of *cecropin A1*, *defensin*, *metchnikowin* and *drsl3* increase at pupariation and are not lost in GF conditions, albeit to different degrees (see also S2 Table). (TIF)

S2 Fig **A)** *drs* expression increases at P0 in the *dl*1 heterozygous mutant to the same degree as in the wild-type suggesting this expression peak is independent of this NF-κB factor. (*lsmeans (pairwise~Genotype|P0) = 0*.*1797; lsmeans (pairwise~Stage|{w-};+;+)<0*.*0001; lsmeans (pairwise~Stage|{w-};dl1/Cyo;+) = 0*.*0001*). **B**) Reduced expression of ecdysone sensitivity in the fat body and haemocytes does not affect the expression of *cecropin A1*, *defensin*, *metchnikowin* and *drsl3* (see also S4 Table). **C)** Reduced ecdysone sensitivity in the haemocytes does not affect the expression of of *cecropin A1*, *defensin*, *metchnikowin* and *drsl3* (*lsmeans(pairwise~Stage|Hml> mcD8GFP)<0*.*0001*, *lsmeans(pairwise~Stage|Hml>EcR DN)<0*.*0001*) (see also S6 Table). **D).** The expression of *cecropin A1*, *defensin*, *metchnikowin* and *drsl3* is unaffected by decreased ecdysone sensitivity specifically in the fat body (see also S7 Table).
(TIFF)

S3 Fig Decreased ecdysone signalling in the midgut results in significant decrease in mRNA levels for: *cecropin A1* (*lsmeans(pairwise~Genotype|P0)<0*.*0001; lsmeans(pairwise~Stage|Mex>EcRDN) = 0*.*0038)*, *defensin (lsmeans(pairwise~Genotype|P0) <0*.*0001; lsmeans(pairwise~Stage|Mex>EcRDN) = 0*.*3866*), *metchnikowin* (*lsmeans (pairwise~Genotype|P0) = 0*.*0060; lsmeans (pairwise~Stage|Mex>EcRDN<0*.*0001*), and *drsl3 ((lsmeans(pairwise~Genotype|P0) = 0*.*0008; lsmeans(pairwise~Stage|Mex>EcRDN) = 0*.*2084*). (TIF)

S4 Fig The expression of *drs* increased at pupariation regardless of the genetic background (*lsmeans(pairwise~Stage|w1118)<0*.*0001; lsmeans(pairwise~Stage|outbred <0*.*0001; lsmeans(pairwise~Stage|Iso)<0*.*0001*). Each dot represents a sample of five pooled individuals; the lines connect the median of the samples at L3 and P0; different letters represent statistically significant differences in fold-change. Fold-change was determined using the ΔΔCT method.
(TIF)

S5 Fig AMP expression upon conditional CRISRP-mediated knock-outs for the common region of *br* and *br-Z4*. Each dot represents a sample of five pooled individuals; the lines connect the median of the samples at L3 and P0; different letters represent statistically significant differences in foldchange (see also S9 Table), determined using the ΔΔCT method. (TIF)

S6 Fig **A)** JASPAR DNA binding motif MA0013.1 for Br-Z4. **B)** Br-Z4 binding motifs predicted by FIMO software (http://meme-suite.org/tools/fimo))[42] to be present in 2kb region of the 5’-end of the *drs* gene, using a p-value<0.0001 as filter.
(TIFF)

S7 Fig **A)** Control ({*w-};+;+*) and *drs* mutant ({*w-};+; Drs r{w+}*) lines showed no differences in mortality between L3, pupae and adults (*lsmeans(pairwise~Stage|Genotype) <0*.*0001;* **B).** Bacteria quantification discriminating *Lactobacillus* and *Acetobacter* in P0 pupae and pharate adults for wild-type and *drs* mutant flies. **C).** Quantification in P0 pupae and pharate adults of *Lactobacillus* and *Acetobacter* without ecdysone signalling in the fat body and haemocytes.
(TIFF)

S8 Fig **A)** Quantification in P0 pupae and pharate adults of *Acetobacter* (MRS medium) and *Lactobacillus* (Manitol medium) without ecdysone signalling in the gut. **B).** Quantification in P0 pupae and pharate adults of *Acetobacter* (MRS medium) and *Lactobacillus* (Manitol medium) upon *drsl2* knock-down (RNAi) in the gut.
(TIFF)

S1 Table MODENCODE expression data (RPKM) for 20 AMP transcripts across different developmental stages. (source Flybase).
(XLSX)

S2 Table Statistical analysis of AMPs expression in an isogenic line (*{w-};+;+*) in normal and germ-free (GF) conditions at L3 and P0 stages. lsmeans, pairwise~Genotype | Stage represents differences within the same stage, between genotypes. lsmeans, pairwise~Stage | Genoype represents the difference between L3 and P0 within each genotype.
(XLSX)

S3 Table Statistical analysis of *drs* expression in isogenic(*{w-};+;+*) and *dif1* mutant lines in normal and germ-free (GF) conditions at L3 and P0 stages. lsmeans, pairwise~Genotype | Stage represents differences within the same stage, between genotypes. lsmeans, pairwise~Stage | Genoype represents the difference between L3 and P0 within each genotype.
(XLSX)

S4 Table Statistical analysis of AMP expression in samples with decreased ecdysone sensitivity in the fat body and haemocytes (*Cg>EcRDN*). lsmeans, pairwise~Genotype | Stage represents differences within the same stage, between genotypes. lsmeans, pairwise~Stage | Genotype represents the difference between L3 and P0 within each genotype.
(XLSX)

S5 Table Statistical analysis of AMP expression in samples with decreased ecdysone sensitivity in haemocytes (*Hml>EcRDN*). lsmeans, pairwise~Genotype | Stage represents differences within the same stage, between genotypes. lsmeans, pairwise~Stage | Genotype represents the difference between L3 and P0 within each genotype.
(XLSX)

S6 Table Statistical analysis of AMP expression in samples with decreased ecdysone sensitivity in the fat body (*AkhR>EcRDN*). lsmeans, pairwise~Genotype | Stage represents differences within the same stage, between genotypes. lsmeans, pairwise~Stage | Genotype represents the difference between L3 and P0 within each genotype.
(XLSX)

S7 Table Statistical analysis of AMP expression in samples with decreased ecdysone sensitivity in the migut (*Mex>EcRDN*). lsmeans, pairwise~Genotype | Stage represents differences within the same stage, between genotypes. lsmeans, pairwise~Stage | Genotype represents the difference between L3 and P0 within each genotype.
(XLSX)

S8 Table Statistical analysis of AMP expression in samples with decreased ecdysone sensitivity in the tracheal system (*btl>EcRDN*). lsmeans, pairwise~Genotype | Stage represents differences within the same stage, between genotypes. lsmeans, pairwise~Stage | Genotype represents the difference between L3 and P0 within each genotype.
(XLSX)

S9 Table Statistical analysis of AMP expression when *br* or *br-Z4* are knocked-out specifically in the fat body and haemocytes. lsmeans, pairwise~Genotype | Stage represents differences within the same stage, between genotypes. L3 is coloured in red and P0 in blue. lsmeans, pairwise~Stage | Genotype represents the difference between L3 and P0 within each genotype.
(XLSX)

S10 Table Statistical analysis of mortality between genotypes, across stages. lsmeans, pairwise~Genotype | Stage represents differences within the same stage, between genotypes. lsmeans, pairwise~Stage | Genotype represents the difference between L3 and P0 within each genotype.
(XLSX)

S11 Table 5’-3’ sequences of primers used for qPCR. (XLSX)

S12 Table 5’-3’ sequences of primers used for tissue specific CRISPR. Guide RNA (gRNA) sequences are represented in capital letters.
(XLSX)

S1 Data Numerical data for every dataset and figure in the manuscript. For qPCRs, each number on the sample name represents a biological replicate. The expression level of each gene in all of these was measured twice.
(XLSX)
