# Supplementary figures and images for: Co-option of immune effectors by the hormonal signalling system triggering metamorphosis in Drosophila melanogaster
Source: PLoS Genet. 2021 Nov 29;17(11):e1009916. doi: 10.1371/journal.pgen.1009916 (PMC8659296; doi:10.1371/journal.pgen.1009916)

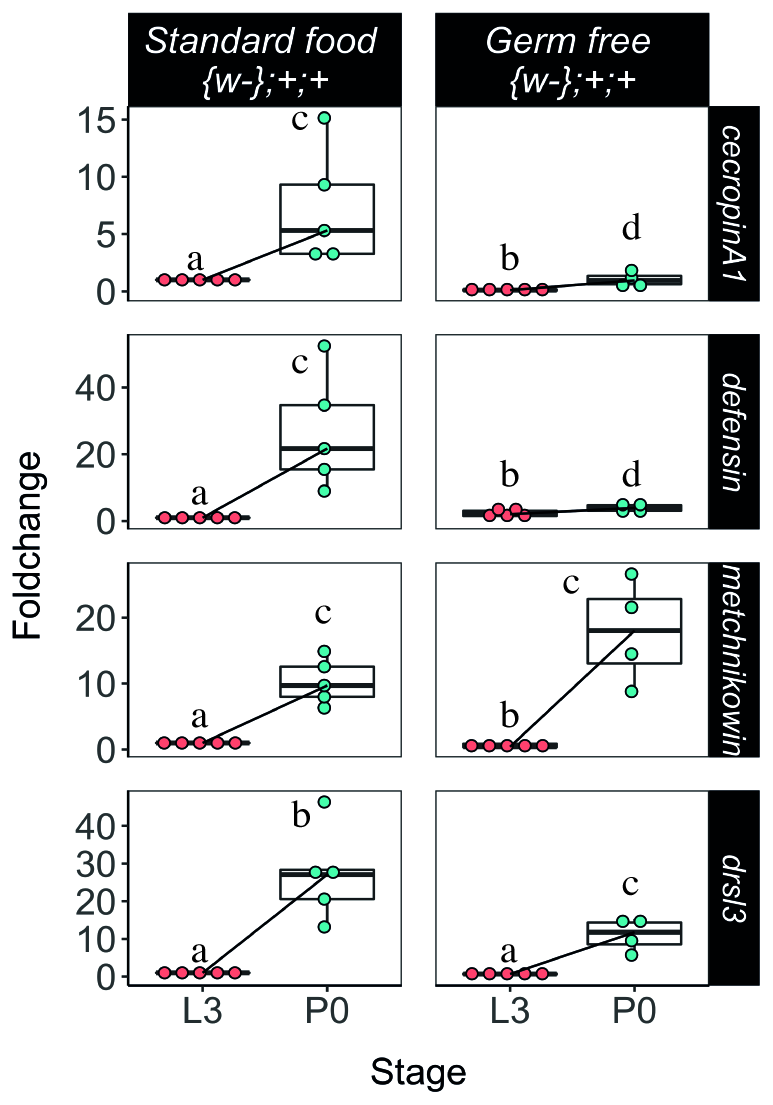

Supplement: S1 Fig — (TIF) [file pgen.1009916.s001.tif]

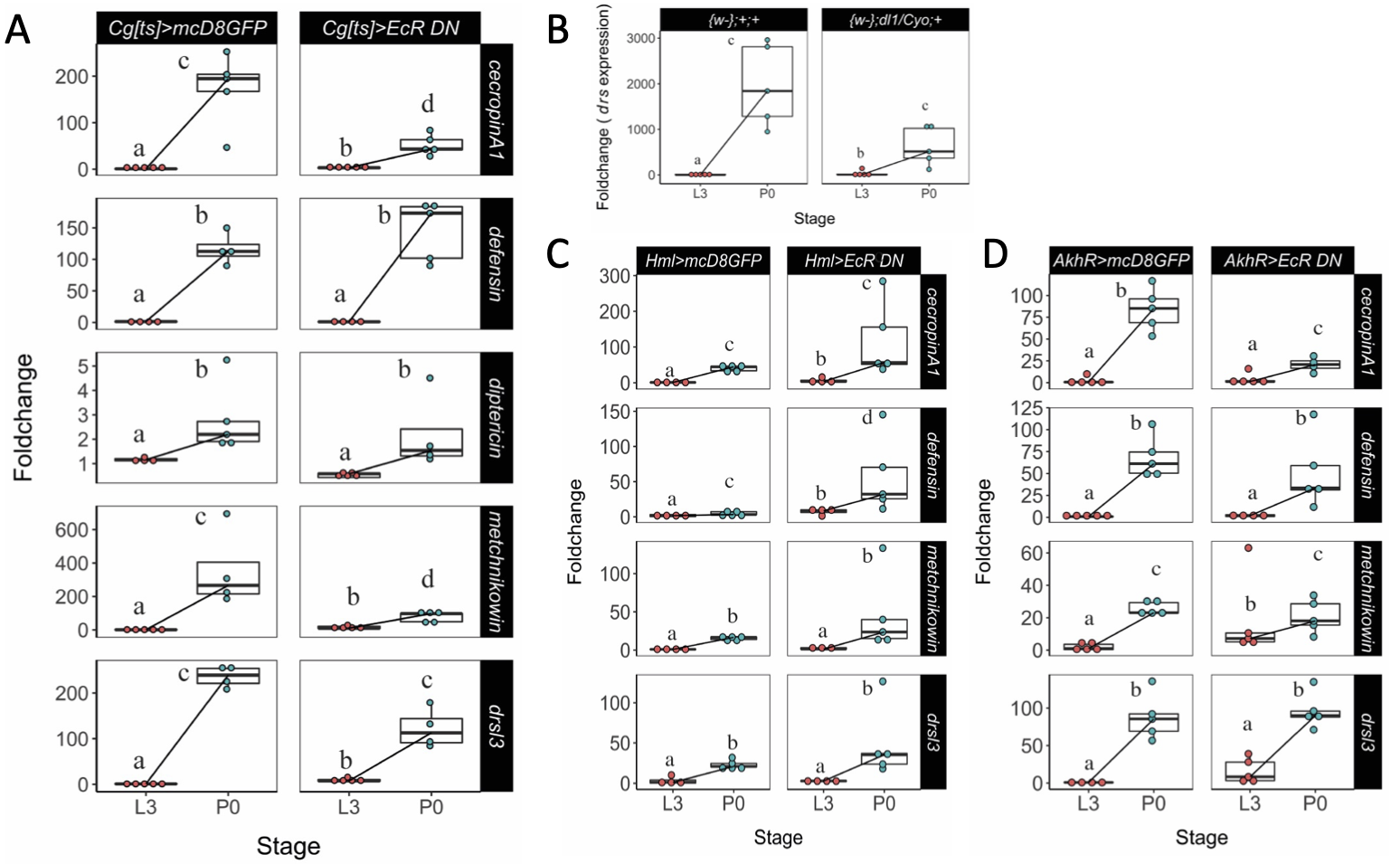

Supplement: S2 Fig — A) drs expression increases at P0 in the dl1 heterozygous mutant to the same degree as in the wild-type suggesting this expression peak is independent of this NF-κB factor. (lsmeans (pairwise~Genotype|P0) = 0.1797; lsmeans (pairwise~Stage|{w-};+;+)<0.0001; lsmeans (pairwise~Stage|{w-};dl1/Cyo;+) = 0.0001). B) Reduced expression of ecdysone sensitivity in the fat body and haemocytes does not affect the expression of cecropin A1, defensin, metchnikowin and drsl3 (see also S4 Table). C) Reduced ecdysone sensitivity in the haemocytes does not affect the expression of of cecropin A1, defensin, metchnikowin and drsl3 (lsmeans(pairwise~Stage|Hml> mcD8GFP)<0.0001, lsmeans(pairwise~Stage|Hml>EcR DN)<0.0001) (see also S6 Table). D). The expression of cecropin A1, defensin, metchnikowin and drsl3 is unaffected by decreased ecdysone sensitivity specifically in the fat body (see also S7 Table). (TIFF) [file pgen.1009916.s002.tiff]

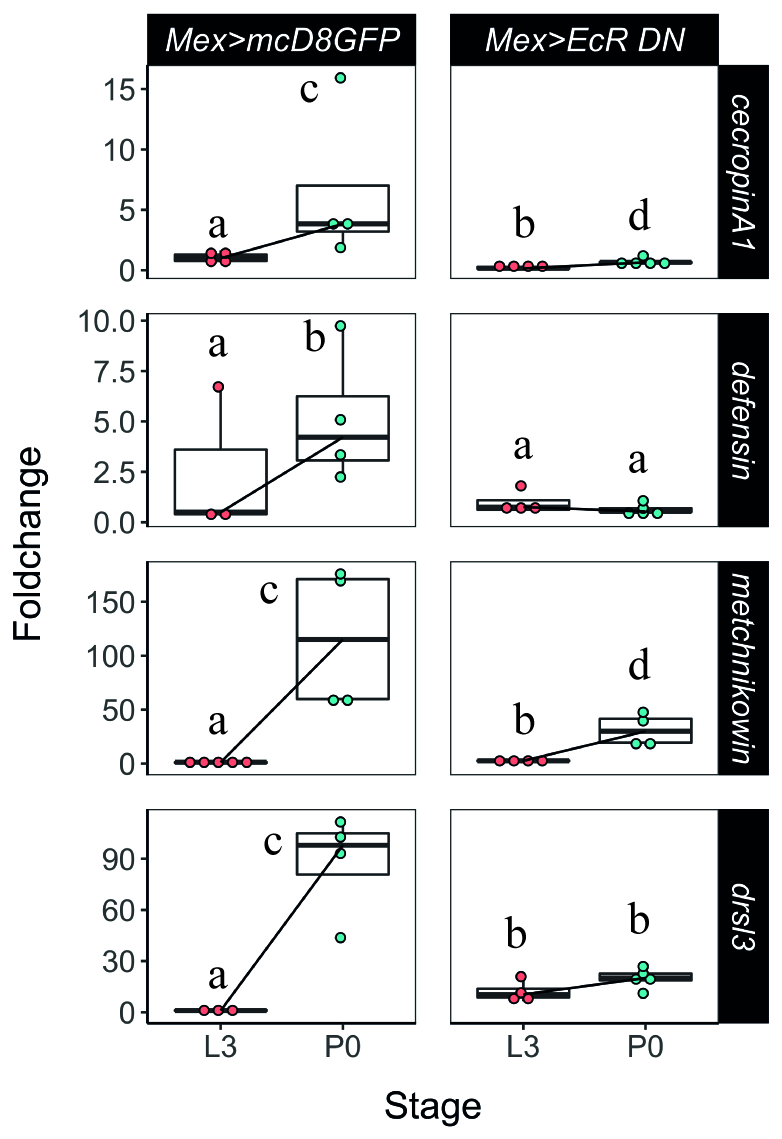

Supplement: S3 Fig — (TIF) [file pgen.1009916.s003.tif]

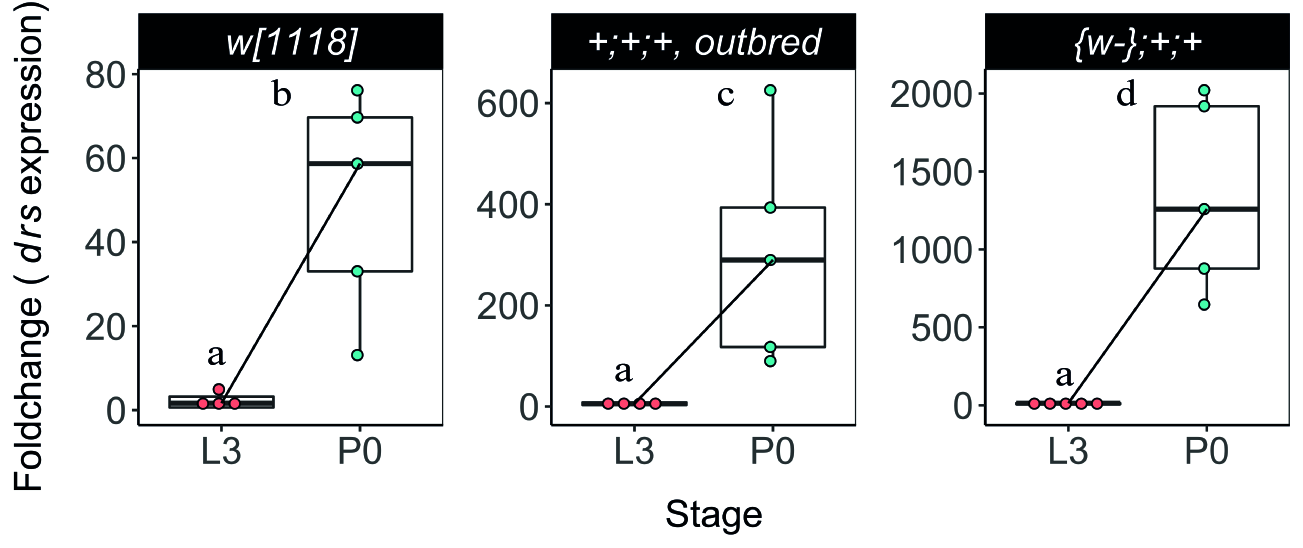

Supplement: S4 Fig — Each dot represents a sample of five pooled individuals; the lines connect the median of the samples at L3 and P0; different letters represent statistically significant differences in fold-change. Fold-change was determined using the ΔΔCT method. (TIF) [file pgen.1009916.s004.tif]

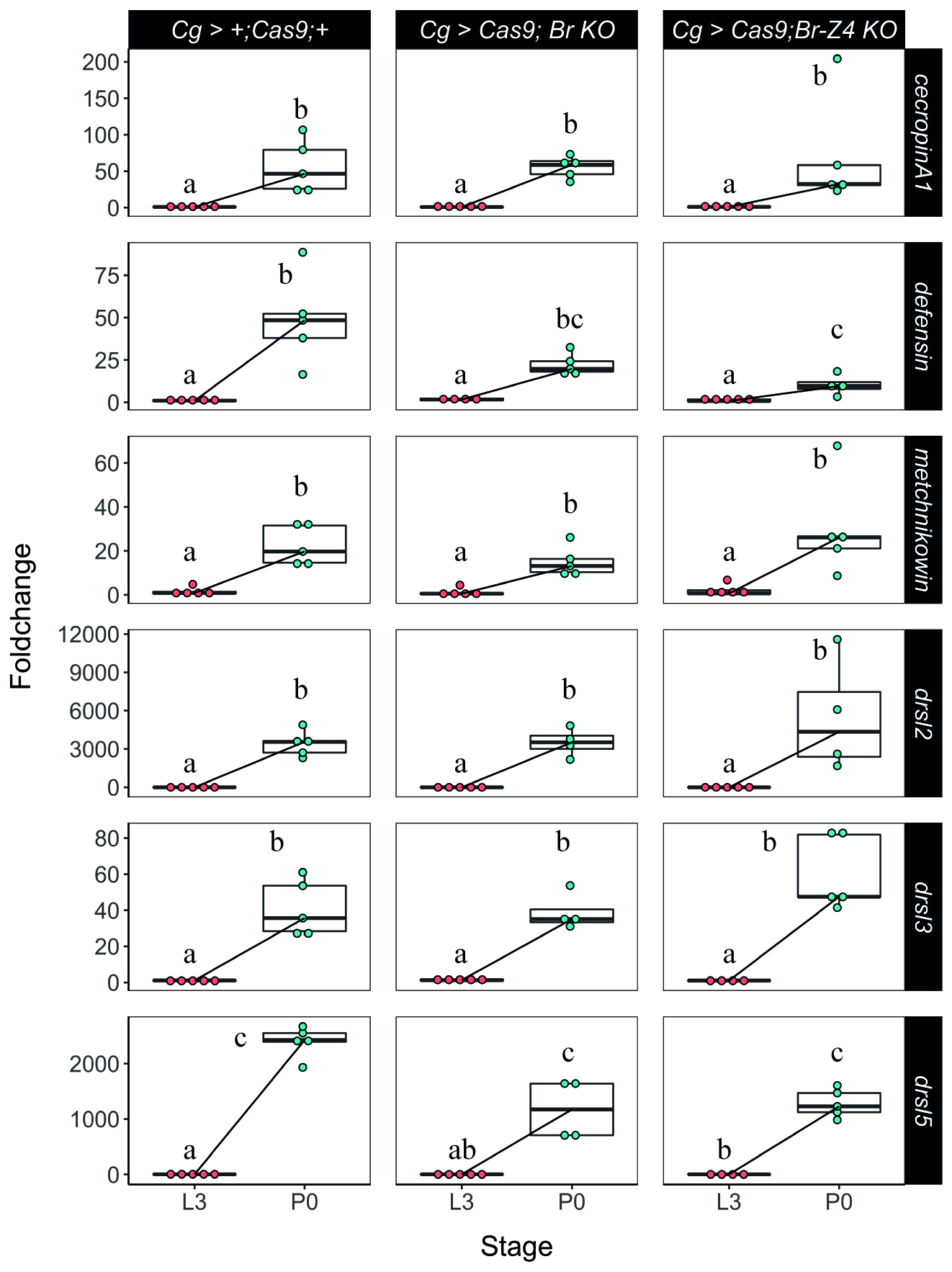

Supplement: S5 Fig — (TIF) [file pgen.1009916.s005.tif]

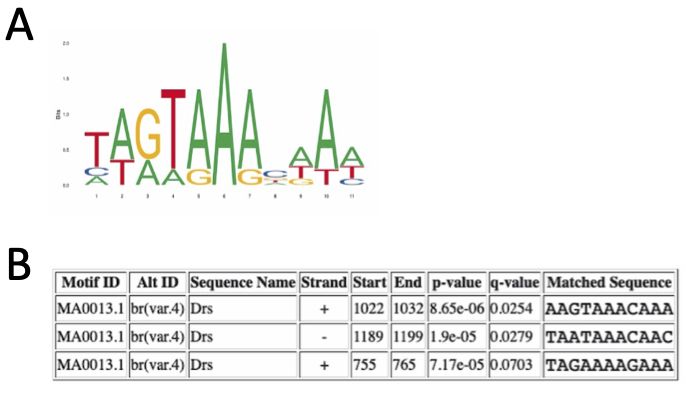

Supplement: S6 Fig — A) JASPAR DNA binding motif MA0013.1 for Br-Z4. B) Br-Z4 binding motifs predicted by FIMO software (http://meme-suite.org/tools/fimo))[42] to be present in 2kb region of the 5’-end of the drs gene, using a p-value<0.0001 as filter. (TIFF) [file pgen.1009916.s006.tiff]

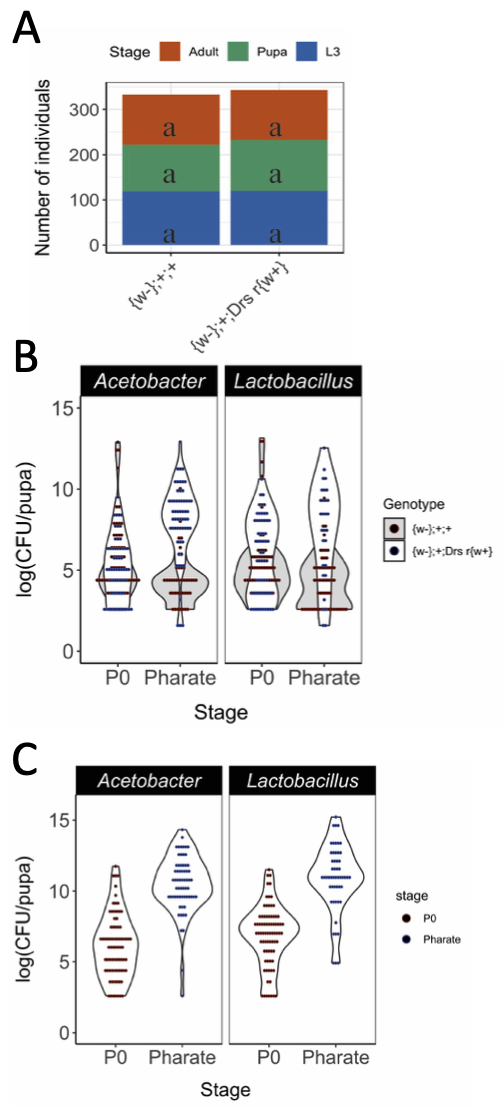

Supplement: S7 Fig — A) Control ({w-};+;+) and drs mutant ({w-};+; Drs r{w+}) lines showed no differences in mortality between L3, pupae and adults (lsmeans(pairwise~Stage|Genotype) <0.0001; B). Bacteria quantification discriminating Lactobacillus and Acetobacter in P0 pupae and pharate adults for wild-type and drs mutant flies. C). Quantification in P0 pupae and pharate adults of Lactobacillus and Acetobacter without ecdysone signalling in the fat body and haemocytes. (TIFF) [file pgen.1009916.s007.tiff]

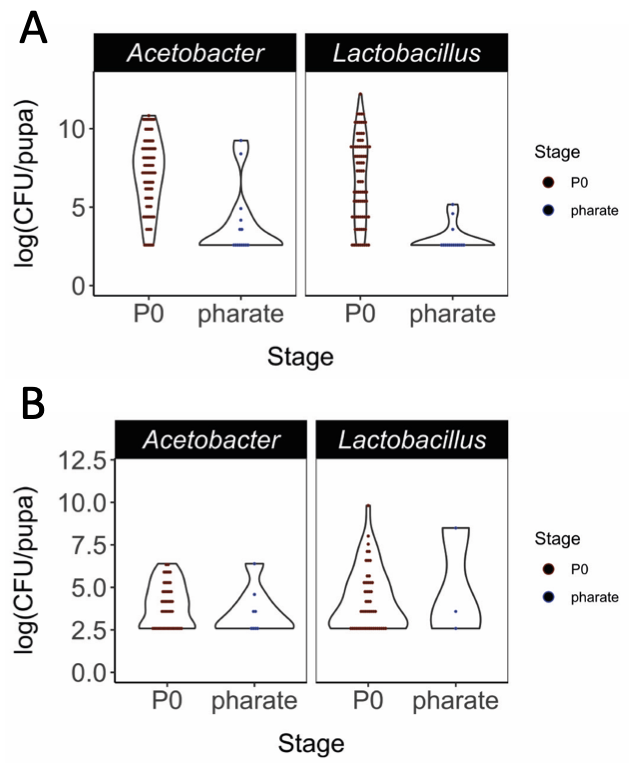

Supplement: S8 Fig — A) Quantification in P0 pupae and pharate adults of Acetobacter (MRS medium) and Lactobacillus (Manitol medium) without ecdysone signalling in the gut. B). Quantification in P0 pupae and pharate adults of Acetobacter (MRS medium) and Lactobacillus (Manitol medium) upon drsl2 knock-down (RNAi) in the gut. (TIFF) [file pgen.1009916.s008.tiff]
